# Mapping genetic markers of artemisinin resistance in *Plasmodium falciparum* malaria in Asia: a systematic review and spatiotemporal analysis

**DOI:** 10.1016/S2666-5247(21)00249-4

**Published:** 2022-03

**Authors:** Frank M Kagoro, Karen I Barnes, Kevin Marsh, Nattwut Ekapirat, Chris Erwin G Mercado, Ipsita Sinha, Georgina Humphreys, Mehul Dhorda, Philippe J Guerin, Richard J Maude

**Affiliations:** a Mahidol Oxford Tropical Medicine Research Unit, Faculty of Tropical Medicine, Mahidol University, Bangkok, Thailand; b WorldWide Antimalarial Resistance Network, Oxford, UK; c Infectious Diseases Data Observatory, Oxford, UK; d Centre for Tropical Medicine and Global Health, Nuffield Department of Medicine, University of Oxford, Oxford, UK; e Collaborating Centre for Optimising Antimalarial Therapy, Division of Clinical Pharmacology, Department of Medicine, University of Cape Town, Cape Town, South Africa; f Harvard T H Chan School of Public Health, Harvard University, Boston, MA, USA; g The Open University, Milton Keynes, UK

## Abstract

**Background:**

The increase in artemisinin resistance threatens malaria elimination in Asia by the target date of 2030 and could derail control efforts in other endemic regions. This study aimed to develop up-to-date spatial distribution visualisations of the *kelch13* (*K13*) gene markers of artemisinin resistance in *Plasmodium falciparum* for policy makers.

**Methods:**

In this systematic review and spatiotemporal analysis we used the WorldWide Antimalarial Resistance Network (WWARN) surveyor molecular markers of artemisinin resistance database. We updated the database by searching PubMed and SCOPUS for studies published between Jan 1, 1990, and March 31, 2021. Articles were included if they contained data on *K13* markers of artemisinin resistance from patients' samples in Asia and articles already included in the WWARN database were excluded. Data were extracted from the published articles and authors were contacted when information was missing. We used the lowest administrative unit levels for the sampling locations of all the *K13* data to describe the spatiotemporal distribution. The numbers of samples tested and those with each molecular marker in each administrative unit level were aggregated by year to calculate the marker prevalence over time.

**Findings:**

Data were collated from 72 studies comprising *K13* markers from 16 613 blood samples collected from 1991 to 2020 from 18 countries. Most samples were from Myanmar (3842 [23·1%]), Cambodia (3804 [22·9%]), and Vietnam (2663 [16·0%]). The median time between data collection and publication was 3·6 years (range 0·9–25·0, IQR 2·7 [2·5–5·2]). There was a steady increase in the prevalence of WHO-validated *K13* markers, with the lowest of 4·3% in 2005 (n=47) and the highest of 62·9% in 2018 (n=264). Overall, the prevalence of Cys580Tyr mutation increased from 48·9% in 2002 to 84·9% in 2018.

**Interpretation:**

From 2002 to 2018, there has been a steady increase in geographical locations and the proportion of infected people with validated artemisinin resistance markers. More consistent data collection, over more extended periods in the same areas with the rapid sharing of data are needed to map the spread and evolution of resistance to better inform policy decisions. Data in the literature are reported in a heterogeneous way leading to difficulties in pooling and interpretation. We propose here a tool with a set of minimum criteria for reporting future studies.

**Funding:**

This research was funded in part by the Wellcome Trust.

## Introduction

Malaria has been declining globally with a 50% reduction of reported cases and an 84% reduction in deaths during the Millennium Development Goals era (2000–15); however, no significant progress in reducing the number of global malaria cases has been made since 2015. Malaria case incidence declined by 27% between 2000 and 2015 and has been increasing since 2015.^1^ Due to the remaining high morbidity and mortality caused by malaria worldwide, global mobilisation efforts have been made for its elimination. The WHO Global Technical Strategy for Malaria 2016–2030 emphasises the goal of eliminating malaria in 35 countries and reducing malaria cases by 90% in malaria-endemic countries compared with 2015.^2^ Effective interventions such as insecticide-treated bednets, indoor residual spraying, rapid diagnostic tests, and more effective artemisinin-based antimalarial treatments coupled with evidence-based targeting have been prioritised.^3^ However, the emergence and spread of artemisinin and artemisinin-based combination therapy partner drug resistance in the Greater Mekong subregion (GMS) pose a substantial threat to these efforts with the potential to spread or emerge further afield, including in to India, Africa, and Latin America.^4^, ^5^, ^6^, ^7^

Effectively managing artemisinin resistance requires tools to monitor its spread over time and space to guide interventions to control or ideally eliminate resistant parasites.^8^ The identification of mutations in the propeller region of the *kelch13* (*K13*) gene associated with artemisinin resistance (with the phenotype of slower parasite clearance) in 2014 has enabled monitoring for artemisinin resistance in research studies and increasingly in routine surveillance.^9^, ^10^, ^11^, ^12^, ^13^, ^14^ Some of the detected mutations are associated with a higher degree of resistance measured as a slower parasite clearance and have a wider geographical distribution than others; for instance, Cys580Tyr is predominant in much of the southeastern GMS.^13^ According to WHO, artemisinin resistance should be suspected in a population if more than 10% of patients are still carrying parasites 3 days after the start of artemisinin or artemisinin-based combination treatment and is confirmed when there is a concurrent validated *K13*-propeller domain mutation present.^14^ The former requires follow-up and retesting of patients; however, this cannot be done in many settings outside of therapeutic efficacy studies in sentinel sites or as part of research studies, all of which are resource-intensive. A practical alternative that is increasingly being used is to collect blood samples as part of routine surveillance to monitor the prevalence of validated *K13* mutations over a large area. Such results can then be used to inform therapeutic efficacy study site selection or trigger a further investigation for studies to identify the resistant phenotype.^11^

**Research in context**

**Evidence before this study**

In 2014, a multicentre study showed the worldwide map of *kelch13* (*K13*) markers and aggregated *K13* markers per country. Additionally, the WorldWide Antimalarial Resistance Network (WWARN) *K13* surveyor charts *K13* global maps with study sites drawn as points using an individual patient data meta-analysis classification. However, no study has aggregated *K13* marker data by provinces or district in a policy maker-friendly format and examined their temporal change in Asia. By March 1, 2021, no such study was obtained from the WWARN database, and from searching PubMed and Scopus databases using the search terms “(artemisinin OR kelch OR kelch13 OR k13) AND (Asia)”.

**Added value of this study**

This is the first study to use the 2020 revised WHO classification for *K13* markers and the first study to aggregate all published *K13* marker data in Asia and to map them by province and district. In this study we also identify that non-uniform, patchy, and delayed reporting are crucial challenges in *K13* surveillance. To optimise *K13* surveillance, this study proposes a new tool for assessing the minimal essential information to assist in pooling *K13* marker data from different studies.

**Implications of all the available evidence**

There is a steady increase in geographical locations and the proportion of malaria infected people with validated artemisinin resistance markers. However, inconsistent data collection and delayed publication hamper our knowledge of the current status, with the available data providing a snapshot of the situation up to four years ago. More consistent and rapid sharing of data from the same areas are needed to map the spread and evolution of resistance to inform policy decisions better.

There is a range of current initiatives monitoring antimalarial resistance using this method, with the main output being maps of resistance marker prevalence.^15^, ^16^, ^17^ These maps are intended for policy makers to convey information on the geographical distribution and temporal changes in resistance; however, the maps are usually produced separately from different projects with patchy information and varied formats designed by the scientists who generated the data. There is a risk of policy makers misinterpreting the data or not using the maps if the data presented are not clear, understandable, and relevant. Therefore, the maps produced must be comprehensive, reliable, timely, and user-friendly for their use as monitoring tools and for their impact on malaria strategy to be maximised.^18^, ^19^

This study aimed to determine the spatial and temporal distribution of the genetic markers of artemisinin resistance in Asia using data from the published literature on *K13* markers, then present this evidence of artemisinin-resistant *Plasmodium falciparum* malaria in a policy maker-friendly format to help guide the planning of malaria control and elimination strategies.

## Methods

### Study design

This study used a sequential mixed-methods design; here, we present the quantitative part that includes a systematic review and descriptive cross-sectional spatiotemporal analysis. The following part used a qualitative end-user usability assessment that comprised interviews and feedback from national malaria programme staff to optimise map presentation for policy makers, which will be published separately. Therefore, this Article presents only the spatiotemporal analysis findings and maps with optimised formats.

### Search strategy and selection criteria

The previously established database of the molecular markers of artemisinin resistance in the WorldWide Antimalarial Resistance Network (WWARN) surveyor (up to July 31, 2019) was combined with a systematic review of all published research papers in PubMed and Scopus using the search terms “artemisinin”, “kelch”, “kelch13”, and “k13” along with geographical terms as shown in appendix 1 (p 3) from Jan 1, 1990, to March 31, 2021.

Citations were downloaded and screened using Mendeley citation software (version 1.19.4). R software (version 3.6), stringr, and tidytext libraries were used to scan the article titles and abstracts to remove duplicates, non-malaria, non-artemisinin related articles, studies that were not using blood samples taken from patients, and citations that were already contained in the WWARN database.^20^ FMK and RJM reviewed the full articles, scanned to assess the article eligibility (article contained Asia-related *K13* markers of artemisinin resistance from patients' samples), and, if eligible, were included for data extraction into an updated *K13* marker dataset. In case of conflicts a third reviewer (KIB) was invited to review and their decision would be final.

### Data analysis

We examined the full texts of the obtained articles to identify the lowest administrative unit levels for the sampling locations. For the papers with this information missing (n=2) we contacted the authors by email to request these details. Where information was provided on imported cases, the probable location of transmission was used. This process made it possible to aggregate data from multiple studies at least at administrative level 1 (provinces or regions) for all countries. We took a consensus decision to aggregate data to a level that can achieve geographically comparable sizes of subnational administrative unit levels between countries. Therefore, studies in China, India, Pakistan, and Myanmar (with large administrative level 1 units) had data aggregated at administrative level 2 (ie, districts in China, India, Pakistan, and townships in Myanmar). In each study and year of data collection, we identified and extracted all evaluated *K13* molecular markers; this included whether *K13* markers were present or absent and the total number of tested samples. The numbers of samples tested and those with each molecular marker in each subnational level were aggregated by year to calculate the marker prevalence over time. The month and year of sample collection were those reported as the end of sample collection for that administrative unit in the relevant paper where this was available (n=47). For those studies without this administrative unit's information, we used the month of the end of the sample collection period (n=11). For those where only the year was provided, we assumed the month to be December (n=8) to give the most conservative (shortest) estimates of the time from sample collection to publication. Validity and risk of bias between studies and in overall data extracted were mitigated by adhering to the study methods (eligibility, data extraction, and analysis).

The final dataset of aggregated molecular marker prevalence was imported for curation into the RStudio development environment (RStudio, Boston, MA, USA) and geocoded to match The Database of Global Administrative Areas (GADM version 3.6).^21^, ^22^, ^23^, ^24^, ^25^ For location names that could not be matched to those in GADM, Google Maps was used as an additional source of geographical information. This spatial dataset was imported to ArcGIS version 10.6.1 (ESRI, Redlands, CA, USA) to produce maps of the spatial and temporal distribution of *K13* markers.

A descriptive spatiotemporal analysis was done to produce thematic maps of the distribution of drug resistance markers and their trends by year. Firstly, we grouped *K13* mutations by their level of evidence for *P falciparum* artemisinin resistance using the WHO classification (2020), with the categories of WHO validated or confirmed, WHO associated or candidate, WWARN associated, not associated, and wild type (appendix 1 p 4).^7^ WWARN-associated mutations are additional single nucleotide polymorphisms (SNPs) associated with prolonging parasite clearance identified in an individual patient data meta-analysis.^22^ We added an unevaluated category to represent *K13* SNPs reported in publications but not yet considered to be validated or associated mutations in the WHO or WWARN categories.^22^ Secondly, we used line plots and Loess regression to evaluate trends at different levels for temporal analysis.^23^ Trends explored included *K13* SNPs and their various classification groups for all of Asia, for the GMS only, and for individual WHO validated markers. For the trend analysis, we only included study areas with more than one timepoint. Thirdly, for spatial analysis, we used aggregated prevalence by administrative unit levels by year to produce thematic maps of drug resistance markers.

### Role of the funding source

The funder of the study had no role in study design, data collection, data analysis, data interpretation, or writing of the report.

## Results

**Figure 1:**
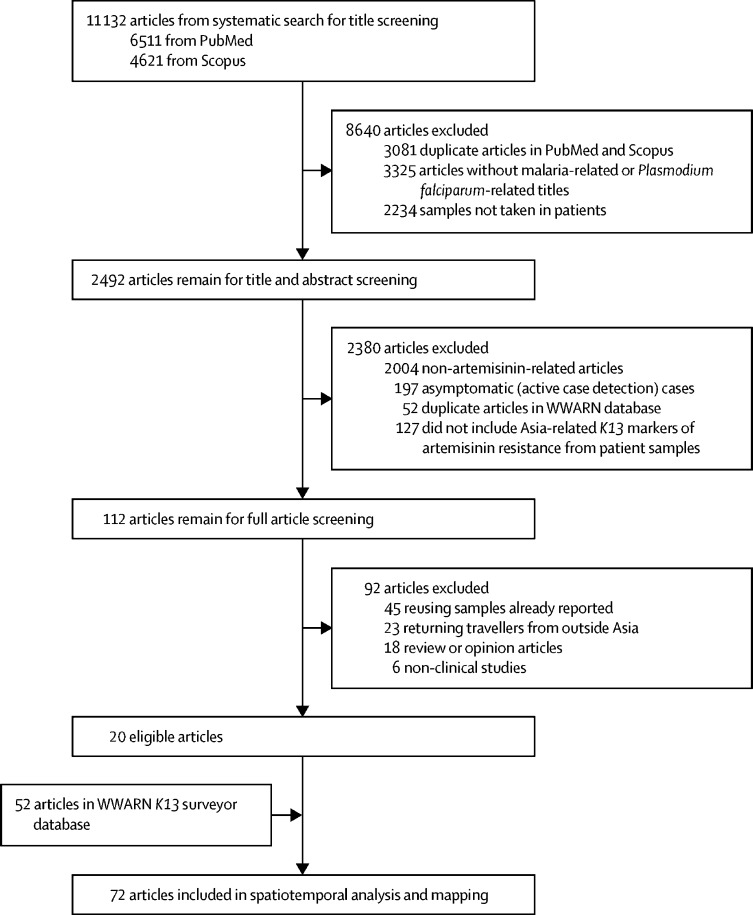
Study selection *K13*=*kelch13*. WWARN=WorldWide Antimalarial Resistance Network.

We obtained 11 132 published papers from the PubMed and Scopus search. 8640 articles were excluded because they were duplicates of articles in the WWARN database, had titles that did not include either malaria-related or *P falciparum*-related information, or did not use blood samples taken from patients. The titles and abstracts of the remaining 2492 articles were screened and 2380 were excluded because they did not mention artemisinin antimalarials, did not have identifying *K13* markers, included samples from asymptomatic patients, and samples collected outside of Asia. Thus, we identified 112 manuscripts for full article review; of these, 92 were excluded (45 re-analysed previously reported data, 23 reported data from returning travellers from outside of Asia, 18 were reviews or opinion articles, and six were non-clinical studies) and 20 were eligible for analysis along with 52 articles in the WWARN database (figure 1). These 72 articles contained data obtained from the analysis of blood samples collected between 1991 and 2020 from both prospective and retrospective (using stored samples) studies. Most of these studies were published between 2015 and 2019, with a mean of 6·2 publications per year (appendix 1 p 5). Over 53% of samples were collected from 2012 to 2015 (9·8% from 2012, 14·4% from 2013, 16·6% from 2014, and 9·2% from 2015). 54 (82·3%) studies were in a single country and 12 were in multiple countries. All studies evaluated molecular markers of artemisinin resistance with some also quantifying therapeutic efficacy using clinical treatment failure rates (n=28), parasite clearance times or rates (n=13), or in vitro phenotype (n=12).

**Figure 2:**
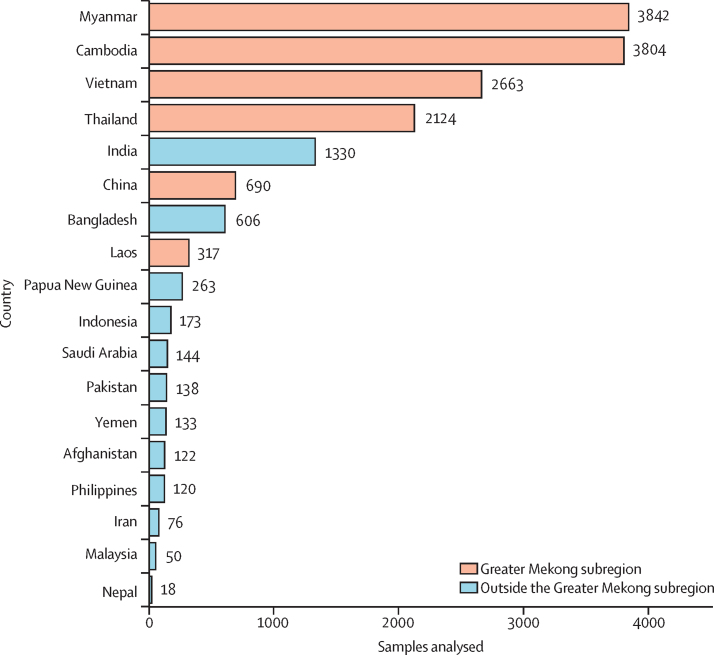
Distribution of samples by country All samples (n=16 613) obtained from the published *K13* studies (n=72) and their distribution, with 13 440 samples from within the Greater Mekong subregion and 3173 samples from outside the Greater Mekong subregion. *K13=kelch13.*

A total of 16 613 samples were collected in 18 different countries in Asia (figure 2). Five studies also evaluated samples from patients who had visited other malaria-endemic countries and were classed as imported cases (n=251). Most of the samples came from the GMS (13 440 [80·9%]), with most from Myanmar (3842 [23·1%]), Cambodia (3804 [22·9%]), Vietnam (2663 [16·0%]), and Thailand (2124 [12·8%]).

The highest numbers of samples analysed by year were in 2014 (2750 [16·6%]) and 2013 (2384 [14·4%]), and few samples were included from before 2009 (appendix 1 p 6). The samples originated from a total of 125 different administrative units (level 2 for China, India, Pakistan, and Myanmar, and level 1 for all other countries). Of these, 60 (48%) administrative units were in the GMS.

The median number of samples collected per administrative unit for all years combined was 42 (range 1–2052), with 40 (32%) administrative units having less than 20 samples and 33 administrative units (26·4%) having more than 100 samples.

The median time from sample collection to publication by the administrative unit was 3·6 years (range 0·9 – 25·0; IQR 2·7 [2·5–5·2]; appendix 1 p 8). Two studies retrospectively evaluated samples collected in 1991 (n=38) and 1997 (n=36) and were published in 2016. We divided the samples into two groups depending on whether samples were collected before or after the discovery of the *K13* marker in 2014.^25^ For samples collected before the discovery, there was a median lag of 4·5 years (range 1·0–25·0; IQR 3·8 [3·5–7·2]). For samples collected after the discovery, this lag was 2·6 years (range 0·9–5·7, IQR 1·0 [2·4–3·4]).

In Asia there were 173 unique *K13* mutations reported (appendix 1 p 14). We categorised these mutations using 2020 WHO criteria: ten solitary SNPs were classified as validated, 13 isolated and two polyclonal SNPs were classified as associated, and one SNP (Ala578Ser) was classified as not associated and wild type.^7^ The other 91 SNPs were considered as unevaluated because they have not yet been classified by WHO or included in the WWARN's list.

**Figure 3:**
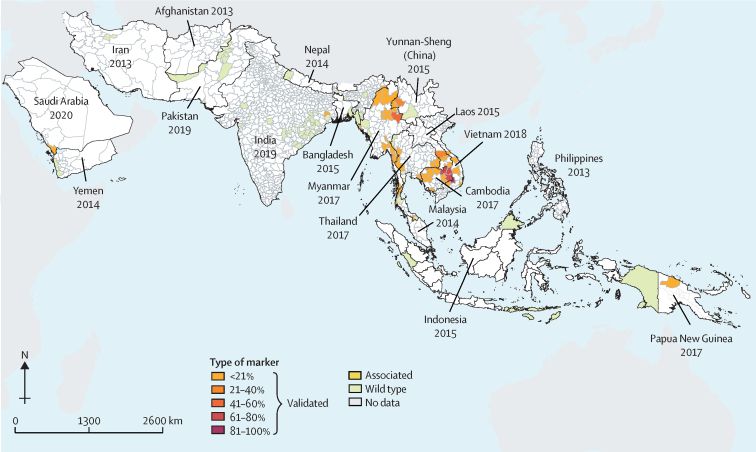
Spatial distribution of *K13* markers in Asia Distribution of the prevalence of *K13* markers and the year of the most recent sample collection for each administrative unit. All molecular markers in that year were aggregated in each administrative unit level 1 (Afghanistan, Bangladesh, Cambodia, Indonesia, Laos, Iran, Malaysia, Nepal, Thailand, Vietnam, and Yemen) and administrative level 2 (China, India, Pakistan, and Myanmar). Validated and associated markers were only found in India and the Greater Mekong subregion. *K13= kelch13.*

Most of the WHO-validated markers of artemisinin resistance were confined to the GMS with the highest prevalence in Cambodia, northeast Thailand, southern Laos, and central Vietnam. These mutations were also identified in three districts in eastern India, one region in Papua New Guinea, and one province in Saudi Arabia. The WHO-associated *K13* markers were found in Cambodia, China, Myanmar, Thailand, and Vietnam. Only wild-type *K13* markers were found in the other locations studied (figure 3).

The two most prevalent validated molecular markers were Cys580Tyr and Phe446Ile (appendix 1 p 10). Most of the cases with Cys580Tyr mutations were found in the southeastern GMS, including Cambodia, Laos, Thailand, and Vietnam. The Phe446Ile mutation was found in the northern GMS, especially at the borders between China and Myanmar, and Thailand and Myanmar. Phe446Ile has been dominant throughout Myanmar except along the border between Myanmar and Thailand, whereby Cys580Tyr was dominant until 2015, following which Phe446Ile predominated. Cys580Tyr was also reported in 2014 and 2017 in Papua New Guinea.

Our study found that samples from the GMS were collected in more years than outside the GMS (appendix 1 p 6). The GMS had the most samples by location with most malaria-endemic areas being covered (appendix 1 p 10). Validated *K13* markers were widely distributed across all the GMS countries with the highest proportions (81–100%) in northeast Thailand, western Cambodia, and central Vietnam. Only associated markers were found in western Myanmar.

Of the 60 administrative units with data in the GMS, 41 (68%) had data from multiple years (appendix 1 p 7). The prevalence of validated markers increased in these 41 locations from 48% to 65% overall from 2002 to 2018. Eight (19·5%) of 41 of these locations included more recent data (from 2015, 2016, 2017, or 2018).

Disaggregated data by country showed Cambodia, Laos, Thailand, and Vietnam to have a clear trend of increased validated markers over time (appendix 1 p 9; video). These trends in China and Myanmar were less clear.

Most bordering regions shared similar proportions of molecular markers**.** We observed the highest prevalence of molecular markers of artemisinin resistance in the eastern parts of the GMS (appendix 1 p 11). The administrative units along international borders had more data and showed varied levels of increase in the prevalence of validated markers over time. This trend was most evident in western Cambodia, eastern Thailand, and southern Myanmar (appendix 1 p 11).

There was a sparse distribution of artemisinin resistance data in India, Afghanistan, Bangladesh, Nepal, and Pakistan (appendix 1 p 13)*.* Although sample locations in India were widely distributed geographically and temporally, with collection years from 2010 to 2019, the number of samples in each district per year was generally lower than in the GMS. Validated *K13* molecular markers were only detected in 2012 and 2015 in India. These markers were found in the districts of Bankura (Arg539Thr; 2015; 8·3% prevalence), Changlang (Arg561His or Arg561Cys; 2012; 4·2% prevalence), and Kolkata (Phe446Ile and Arg539Thr; 2015; 3·6% prevalence). In 2016 and 2019, only *K13* wild-type parasites were reported from 16 districts in India, and in Afghanistan, Bangladesh, and Nepal.

**Figure 4:**
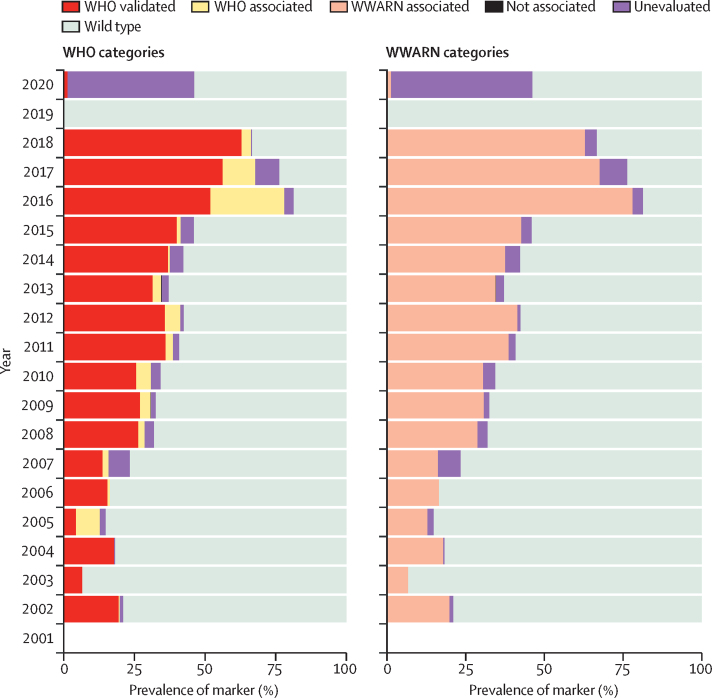
Prevalence of *K13* markers by year All *K13* molecular markers were grouped by category and year independently using two classifications (WHO and WWARN) across all countries. The overall prevalence of each category of molecular markers by year was evaluated. Except for 2019 and 2020, there was a consistent increase of WHO-validated, WHO-associated, and WWARN-associated markers over time. Samples for 2019 (from India and Pakistan) showed wild type parasites, and 2020 samples (from Saudi Arabia) had one WHO-validated mutation (Met476Ile), unevaluated markers, and wild type parasites. WWARN=WorldWide Antimalarial Resistance Network. *K13= kelch13.*

Overall, we found an increase in the prevalence of WHO-validated and WHO-associated markers from a mean of 18·6% for samples collected before 2011 (the minimum of 6·7% in 2003 and maximum of 30·9% in 2010; n=3324) to a mean of 52·9% for 2011–18 (minimum of 34·5% in 2013 and maximum of 87·4% in 2016; n=12 577; figure 4). The increase in the prevalence of WWARN-associated markers had a mean of 20% before 2011 and 52·5% between 2011 and 2018 for the same samples. The prevalence of the validated markers increased consistently throughout the study period, with the lowest of 4·3% in 2005 (n=47) and the highest of 62·9% in 2018 (n=264). Only two studies had samples for 2019 (India and Pakistan) and one for 2020 (Saudi Arabia). All the 2019 samples had wild-type parasites (n=202), and 2020 samples had one WHO-validated mutation (Met476Ile), unevaluated markers, and wild-type parasites. All of the 2020 samples were reported in Jizan Province in Saudi Arabia (n=80). None of the GMS administrative units had samples after 2018.

In the GMS, proportions of cases with validated *K13* mutations increased from 2002 to 2018 and followed a similar pattern over time (appendix 1 p 12). For associated and unevaluated markers, 34 (57%) of the 60 administrative units collected samples in only 1 year. The median of the number of years with samples from the same administrative unit was 1·0 year (range 1·0–14·0; appendix 1 p 7). Only 52 (41·6%) of all administrative units in Asia (including 13 157 [79·2%] of all samples collected and 41 [68·3%] of 60 administrative units with data in the GMS) had samples from more than 1 year so could be included in trend analyses.

Overall, the prevalence of WHO-validated molecular markers has increased in the GMS. This increase in prevalence was slow before 2008, levelled off from 2011 to 2014, and then accelerated from 2015 onwards (appendix 1 p 12). The amount of data varied over time, with fewer geographical locations covered before 2010 and in 2018. The changes over time in the WHO-validated *K13* marker category was similar to the WWARN-associated markers presented in the WWARN individual patient data meta-analysis; there was also a slight increase of WHO-associated and unevaluated markers from 2010 to 2015 (appendix 1 p 12).^22^

**Figure 5:**
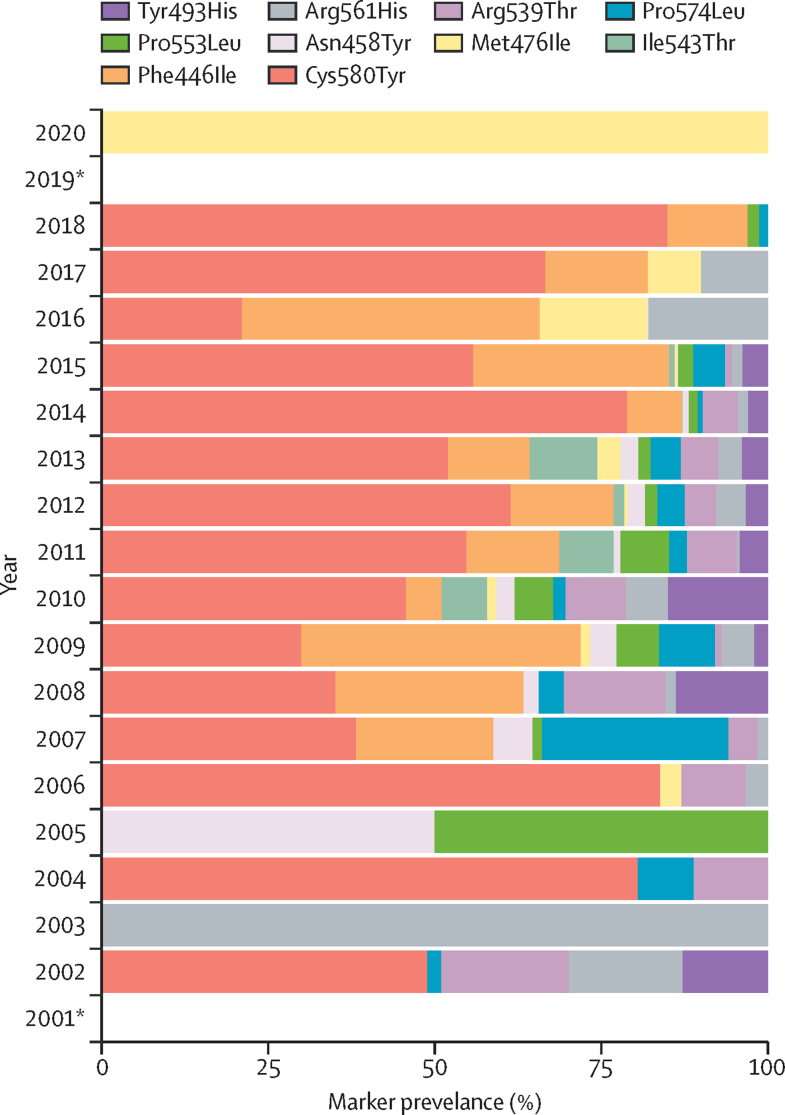
Temporal trends of individual WHO-validated markers All samples with single nucleotide polymorphisms categorised as WHO validated pooled together by year and their overall proportions. The 2019 and 2020 samples all came from outside the Greater Mekong subregion, with the result for 2020 being from one (1%) of 80 blood samples from Saudi Arabia. Excluding 2019 and 2020, the Cys580Tyr mutation was the most common WHO-validated mutation in almost every year except for 2003 and 2005. *No validated markers reported.

Relatively fewer samples were published for 2019 and 2020 than for other years, no validated mutations were reported in 2019, and only one sample had the Met476Ile marker (n=80). Before 2019, the Cys580Tyr marker was detected every year, except in 2003 and 2005 (figure 5). Some with Cys580Tyr were listed as Cys580Tyr/Cys, which indicates parasites with both Cys580Tyr and wild type (ie, polyclonal infection). There was an increase in samples with Cys580Tyr over time, accounting for most samples in all years except for 2003 and 2005. There was an increase in the overall proportion of samples with Cys580Tyr or Cys580Tyr/Cys, or both, from an mean of 42·4% between 2002–06 to 71·8% of samples between 2014–18. The prevalence of Cys580Tyr mutation increased from 48·9% in 2002 to 84·9% in 2018.

## Discussion

We have presented a description of all currently available information on changes in the geographical distribution of genetic markers of *P falciparum* artemisinin resistance markers (*K13* mutations) over time in Asia, including the most recent published data and a subset of unpublished data (up to July, 2020). To do this analysis, we searched citations from the PubMed database and Scopus to update the WWARN *K13* dataset. We added additional spatial and temporal details to produce a dataset of *K13* markers containing 72 citations. The *P falciparum K13* propeller mutations were used to approximate the spatiotemporal distribution of artemisinin resistance, with an increase in resistance markers by type and geographical extent shown in a range of thematic maps. Over time, we observed a consistent reporting of an increase in the prevalence of artemisinin resistance validated and associated markers in Asia. These mutations were first identified along the border between Cambodia and Thailand in the Battambang and Pailin provinces.^26^, ^27^ By 2009, validated and associated mutations had only been identified in three countries, Cambodia, Myanmar, and Thailand. By 2018, this number had increased to include six additional countries (China, India, Laos, Papua New Guinea, Saudi Arabia, and Vietnam). Thus, increases in the prevalence and extent of WHO-validated or WHO-associated markers over time were seen in Myanmar, Thailand, Cambodia, Vietnam, Laos and, to a lesser extent, in China. Except for India and Papua New Guinea, most of the locations with marker data were along international borders, particularly in the GMS. These border areas are generally where *P falciparum* malaria is most prevalent in these countries.^28^

In the present study, we found that although the Cys580Tyr mutation occurs in all the GMS countries, the most frequent validated mutations in the eastern GMS (Cambodia, Laos, east Thailand, and Vietnam) were Cys580Tyr, Arg539Thr, Tyr493His, and Ile543Thr. In western GMS (China, Myanmar, and west Thailand), the most prevalent validated mutations were Phe446Ile, Asn458Tyr, Pro574Leu, and Arg561His. This distribution is consistent with previously published studies, showing localisation of mutations in specific geographical areas, such as Asn458Tyr and Arg561His in the eastern and northern GMS, Arg539Thr in southern and east GMS, and Ile543Thr and Tyr493His in east GMS.^15^ Such distribution supports the theory of independent emergence of artemisinin resistance in several locations, with Cys580Tyr sweeping through the region.^29^, ^30^ On the border between Thailand and Myanmar, Cys580Tyr predominated until 2016, when Phe446Ile became more prevalent. This change coincided with an intense effort to eliminate *P falciparum*, which resulted in a significant decrease in cases.^31^ Treatment regimens might need to be adjusted for locations with a high prevalence of the subset of *K13* markers that are known to be associated with markedly slower parasite clearance and of markers of resistance to artemisinin and artemisinin-based combination therapy partner drugs.^32^

Our analysis found a long lag time between sample collection and publication (median 3·6 years). Part of this lag was due to many studies retrospectively analysed samples that had already been collected before the discovery of the *K13* marker in 2014. For studies only analysing samples collected after the discovery of *K13*, the lag was still substantial at 2·6 years, despite using conservative assumptions for studies in which detailed time information was not available. This lag limits the usefulness and relevance of this marker data for guiding policy decisions. The relative lack of published studies reporting the prevalence of *K13* markers in samples collected in 2019 and 2020 is likely to be due to this lag between sample collection and publication. The absence of validated or associated *K13* markers in those years is therefore a consequence of the published data being from only a few sites in Pakistan, India, and Saudi Arabia. Large scale sample collections have been ongoing in the GMS during these years, which will contribute more *K13* distribution data in the near future.^33^

This study has several limitations. Samples were pooled from different studies with different study designs, durations, and sample sizes. Our analysis was limited to published studies and a subset of unpublished data shared and displayed on the WWARN Artemisinin Molecular Surveyor. There were inevitable differences in the level of detail reported and available for each study, particularly concerning which *K13* mutations were investigated (and which were not) and when and where the samples were collected. This disparity required us to either make assumptions or constrain the possible spatial and temporal resolution of the analysis. Because of these limitations, we propose a set of reporting criteria for studies that collect *K13* molecular marker data, and we have developed a tool that can be used to collate data from published studies and generate a score for these minimal criteria (appendix 2).

The high disparity of data across the region over time and across geographical locations limited our ability to describe the spatial and temporal trends fully. Only 41·6% of the 125 administrative units included had samples from at least two different timepoints. Thus, it was not possible to assess the trend in resistance marker prevalence for more than half of the administrative units and seven entire countries (Afghanistan, Iran, Malaysia, Nepal, Pakistan, Philippines, and Yemen) with data. Although the *P falciparum* malaria-endemic areas of the GMS were relatively well represented, with 84·6% of samples collected in administrative units, with repeated sampling, the data from other countries were sparser both in space and time. The lack of published *K13* markers from some areas might have been due to fewer studies being done in those areas, delayed reporting, or no reporting. We also noted that the *K13* markers from the GMS area were only present until 2018 as there was no published study with samples collected after that year.

Prompt reporting of routine surveillance or research activities with a consistent, repeated collection of molecular markers in the exact location (sentinel sites) would allow for more accurate and informative spatial and temporal analysis. The present study's findings can help identify those areas that would be most suitable for such surveillance activities. However, unless sentinel sites have broad coverage, detecting the emergence of resistance might also be delayed. This study used the *K13* propeller mutations to describe artemisinin resistance; however, a 2017 study suggests other *K13* mutations apart from propeller mutations (eg, Glu252Gln, Asp281Val, and Arg239Gln) might also be associated with artemisinin resistance.^34^ Because the evidence for these markers is absent or scarce due to them rarely being tested for, *K13* propeller mutations remain the preferred marker for surveillance of *P falciparum* artemisinin resistance. Our study found a considerable number of unevaluated SNPs that have not yet been classified or graded by WHO or included in the WWARN individual patient data meta-analysis. Such markers could potentially influence the artemisinin efficacy in the geographical areas analysed in this study. Delays in evaluating the association of molecular markers with delayed parasite clearance contribute to the increase of unevaluated SNPs; to date there has been a 3-year gap between WHO evaluations (2014, 2017, and 2020) and only a single evaluation by WWARN in 2017.

This study combined data from all available published sources to define the increase in prevalence and geographical extent of artemisinin resistance markers in Asia. More consistent data collection is needed from the exact locations, from a wider geographical area and over more extended periods to map the spread and evolution of resistance as it continues to unfold. This study also highlights the need for more rapid dissemination of molecular marker data so it is available in time to guide policy decisions.

## Data sharing

The complete and updated datasets, plots, and maps generated and analysed during this study are publicly available on the WWARN website (https://www.wwarn.org/tracking-resistance/artemisinin-molecular-surveyor).

## Declaration of interests

We declare no competing interests.

## Supplementary Materials

Supplementary appendix 2

Supplementary Video Temporal and spatial trends of kelch13 (K13) markers in the Greater Mekong subregion This graphic interchange file displays the change of the overall prevalence of validated molecular markers by administrative unit over time from published K13 studies in the Greater Mekong subregion. All countries show an increase in the validated K13 markers. **YouTube URL:**https://youtu.be/_d0dneGA1Hg
