# Supplementary material for: Mapping genetic markers of artemisinin resistance in Plasmodium falciparum malaria in Asia: a systematic review and spatiotemporal analysis
Source: Lancet Microbe. 2022 Mar;3(3):e184–92. doi: 10.1016/S2666-5247(21)00249-4 (PMC8891024; doi:10.1016/S2666-5247(21)00249-4)
Supplement: Supplementary appendix 1 [file mmc1.pdf]

THE LANCET
Microbe

Supplementary appendix 1

This appendix formed part of the original submission and has been peer reviewed. We post it as supplied by the authors.

Supplement to: Kagoro FM, Barnes KI, Marsh, K, et al. Mapping genetic markers of artemisinin resistance in *Plasmodium falciparum* malaria in Asia: a systematic review and spatiotemporal analysis. *Lancet Microbe* 2022; published online Jan 25. https://doi.org/10.1016/S2666-5247(21)00249-4.

Appendix 1

Study title

Charting the enemy lines - mapping genetic markers of artemisinin resistance in Plasmodium falciparum malaria in Asia: a systematic review and spatiotemporal analysis

Authors

Frank M. Kagoro MSc [1,2,3,4,5], Prof Karen I. Barnes MMed[2,5], Prof Kevin Marsh MBChB[4], Nattwut Ekapirat MSc[1], Chris Erwin G. Mercado MSc[1], Ipsita Sinha MSc[1,4], Georgina Humphreys PhD[2,4], Mehul Dhorda PhD[1,2,3,4], Prof Philippe J Guerin MD[2,3,4], Prof Richard J. Maude DPhil[1, 4, 6, 7]

First author

Dr Frank M. Kagoro
M.D., PgD HCTMC, MSc
PhD Fellow, University of Cape Town, South Africa
frank.kagoro@ndm.ox.ac.uk | M +27 795920379
Research Physician, Epidemiology Department, Mahidol-Oxford Tropical Medicine Research Unit, Faculty of Tropical Medicine, Mahidol University & Infectious Diseases Data Observatory (IDDO), Centre of Tropical Medicine and Global Health, University of Oxford

Corresponding author

Professor Richard J Maude
MBChB Hons BSc DTM&H DPhil MD FRGS FRCP FRSPH
Head of Epidemiology Department, Mahidol-Oxford Tropical Medicine Research Unit, Faculty of Tropical Medicine, Mahidol University, Bangkok 10400, Thailand
richard@tropmedres.ac | T 662 203 6333 | F 662 354 9169
Professor of Tropical Medicine, Centre for Tropical Medicine and Global Health, Nuffield Department of Medicine, University of Oxford, Oxford, OX3 7FZ, UK
Visiting Professor, The Open University, Milton Keynes, MK7 6AA, UK
Visiting Associate Professor, University of Hong Kong (HKU), Hong Kong
Visiting Scientist, Harvard TH Chan School of Public Health, Harvard University, Boston, MA 02115, USA

Contents

Table S1: Search strategy 3
Table S2: Classification of K13 mutations 4
Table S3. Included K13 studies by publication year 5
Figure S1: Distribution of samples by year 6
Table S4. Years of sample collection by administrative units 7
K13 markers publication lag 8
Figure S2: Interval between sample collection and publication 8
Figure S3. Difference between sample collection and publication time 8
Figure S4. Temporal trends of K13 markers per country 9
Figure S5: WHO-validated markers in the GMS 10
Distribution of K13 markers in the GMS 11
Figure S6: Trend of WHO-Validated markers in selected locations in the GMS 11
Figure S7. Temporal and spatial trends of K13 markers in the GMS (additional MS Power Point file) 11
Figure S8: Temporal trends of K13 marker prevalence in the GMS 12
Figure S9: Distribution of K13 markers in South Asia 13
Table S5. K13 mutations by category. 14
Tool S1. The proposed K13 marker study reporting criteria (additional excel file). 15
Table S6. Kelch 13 markers publications. 16

Table S1: Search strategy

#1	artemisinin OR kelch OR kelch13 OR k13
#2	Asia OR Greater Mekong OR Bangladesh OR Cambodia OR China OR Indonesia OR Malaysia OR Myanmar OR Lao OR Afghanistan OR Viet Nam OR India OR Iran OR Nepal OR Philippines OR Thailand OR Bhutan OR Oman OR Lebanon OR Jordan OR United Arab Emirates OR Yemen OR Syria OR Kyrgyzstan OR Mongolia OR Iraq OR Saudi Arabia OR Taiwan OR Uzbekistan OR Turkmenistan OR Tajikistan OR Kazakhstan OR Azerbaijan OR Russia OR Armenia
#3	#1 AND #2

Table S2: Classification of K13 mutations

Categories	SNPs	Meaning
**WHO validated/ confirmed**	C580Y, F446I, I543T, M476I, N458Y, P553L, P574L, R539T, R561H, Y493H	A SNP with evidence of correlation with artemisinin efficacy decline in clinical studies and in vitro studies and included in the list of WHO validated mutations.
**WHO associated**	A481V, A675V, C469F, C469Y/F, E252Q, F673I, G449A, G449A/D, G538V, N537I, P441L, P527H, P574L, R515K, V568G	A SNP with evidence of correlation with a therapeutic decline in clinical studies but without evidence of being tested in in vitro studies and included in the WHO candidate/associated mutations.[24]
**WWARN associated**	***P553L**, +**P441L**, ***F446I**, +**N537I**, ***P574L**, ***R561H**, ***C580Y**, ***Y493H**, **V568G**, ***I543T**, ***R539T**, **R539R/T**, C580C/Y, +**N525D**, ***N458Y**, ***M476I**, +**G538V**, F614L, E252Q, +**A675V**, +**A481V**, H719N, +**G449A/D**, R561H/C, D584V	A SNP with evidence of correlation with slow parasite clearance using the WWARN in vivo parasite clearance half-life estimator.[22]
**Unevaluated**	Any reported K13 SNPs to be determined in the review	A reported K13 SNP but not yet included as a validated/associated mutation by WHO or WWARN.
**Not associated**	A578S	A SNP not associated with a therapeutic decline in either clinical or in vitro studies.
**Wild type**	Wild type SNPs	A non-mutated allele that has full sensitivity to artemisinins.

*Grouped Single Nucleotide Polymorphisms (SNPs) based on their association with P. falciparum clearance rate. The grouping is as per the WHO classification and the published WWARN Individual Patient Data (IPD) meta-analysis (Bolded K13 SNPs show where they mirror the *WHO validated and +WHO associated groups). Unevaluated markers will include all new and old SNPs that have not yet been evaluated to be included in the WHO or WWARN lists.*

Table S3. Included K13 studies by publication year

Year	Total	Cum. Total
2020	8	72
2019	13	64
2018	5	51
2017	14	46
2016	14	32
2015	12	18
2014	5	6
2010	1	1

Table displaying the number of manuscripts included in the review by year of publication. The majority of these studies were published between 2015 and 2019, with an average of 6·2 publications per year overall.

Figure S1: Distribution of samples by year

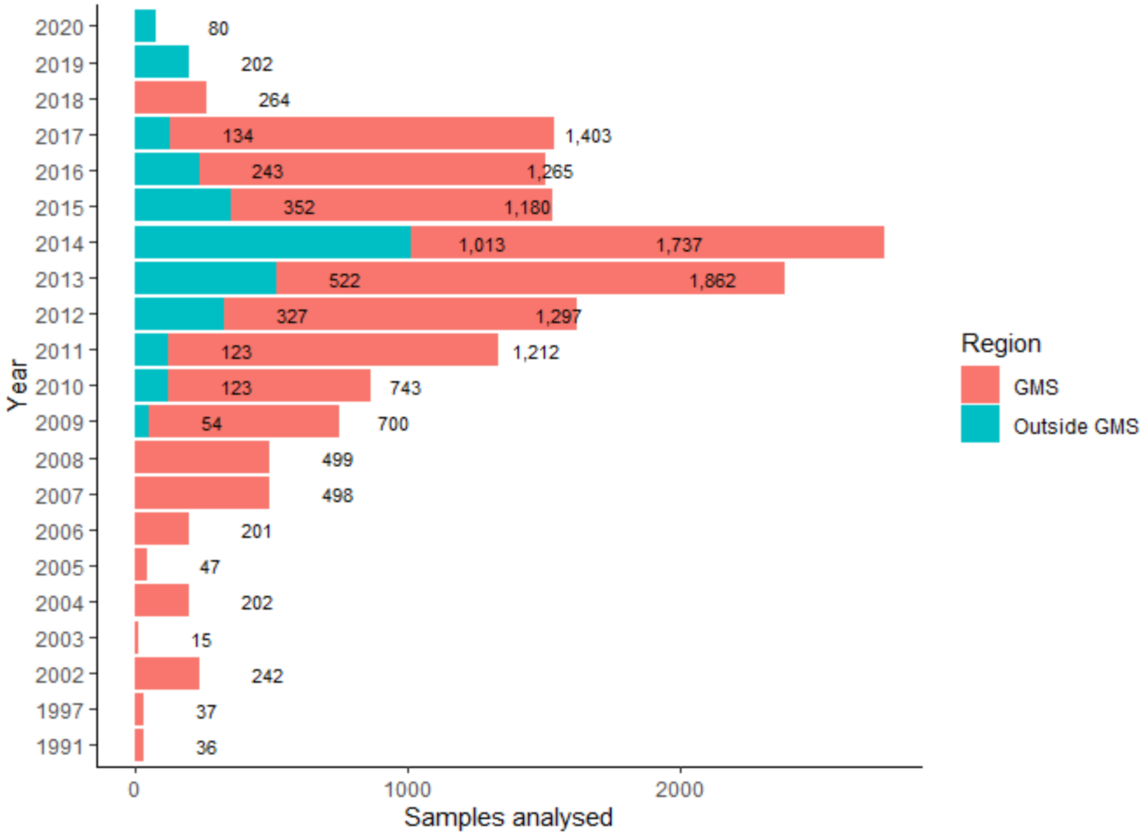

All samples (n=16,613) categorised by year of sample collection and indicating those from the Greater Mekong Subregion (GMS) (n=13,440) and outside the GMS (n=31,73). Of all years, 2014 had the highest number of samples.

Table S4. Years of sample collection by administrative units

	All			GMS			Outside GMS		
Number of years	Admin units	%	Cum. %	Admin units	%	Cum. %	Admin units	%	Cum. %
1	73	58·4	58·4	19	31·7	31·7	54	83·1	83·1
2	21	16·8	75·2	15	25	56·7	6	9·2	92·3
3	9	7·2	82·4	5	8·3	65	4	6·2	98·5
4	5	4	86·4	5	8·3	73·3	0	0	0
5	8	6·4	92·8	7	11·7	85	1	1·5	100
6	2	1·6	94·4	2	3·3	88·3	0	0	0
8	1	0·8	95·2	1	1·7	90	0	0	0
9	3	2·4	97·6	3	5	95	0	0	0
10	1	0·8	98·4	1	1·7	96·7	0	0	0
11	1	0·8	99·2	1	1·7	98·4	0	0	0
14	1	0·8	100	1	1·7	100	0	0	0

Number of years with samples by administrative unit overall, in the GMS and outside the GMS (Admin=administrative, Cum=cumulative). Around 60% of the administrative units had data only for one year. Over 68·3% (n=44) of administrative units had samples from more than two years coming from GMS.

K13 markers publication lag

Figure S2: Interval between sample collection and publication

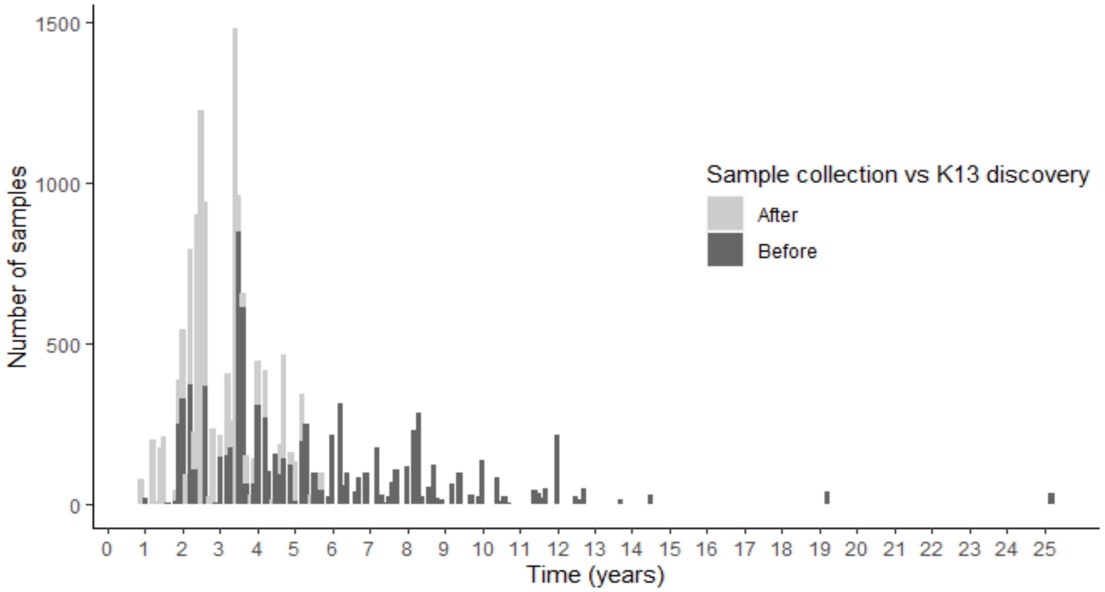

The duration between sample collection and publication calculated as the difference between the two respective dates for each sample. The median (range) time between sample collection and publication was 3·6 (range of 1 to 25 and IQR of 2·7 with the 25th and 75th percentiles of 2·7 and 5·2 respectively) years. Samples collected after the discovery of K13 had shorter interval compared to the rest of the samples.

Figure S3. Difference between sample collection and publication time

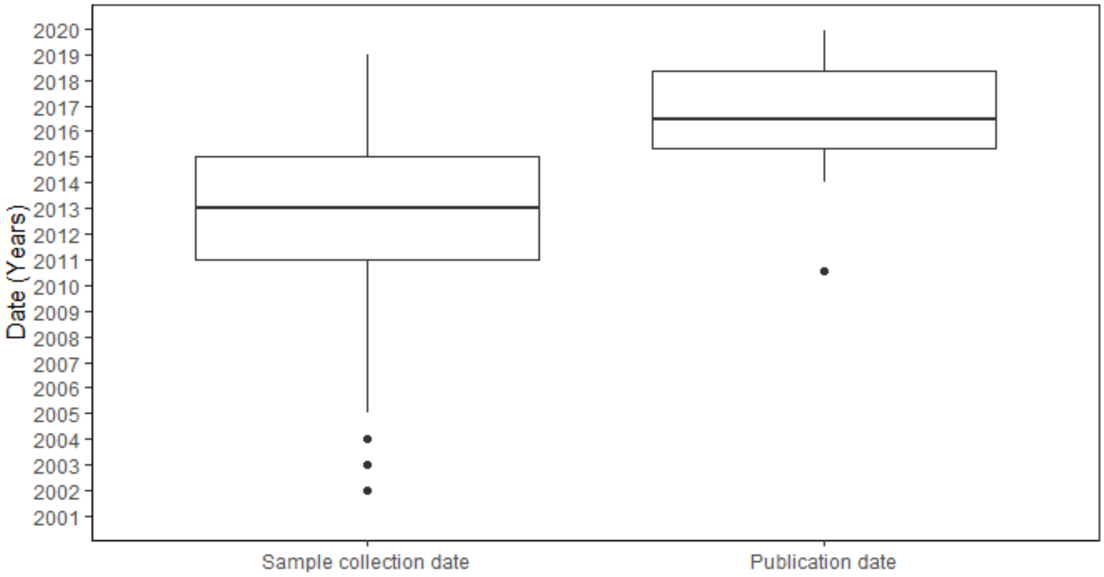

Collection and publication dates, median and interquartile ranges for pooled K13 sample collection and publication dates. The median time between sample collection and publication was 3·6 (range of 1 to 25 and QR of 2·7 with the 25th and 75th percentiles of 2·7 and 5·2 respectively) years.

Figure S4. Temporal trends of K13 markers per country

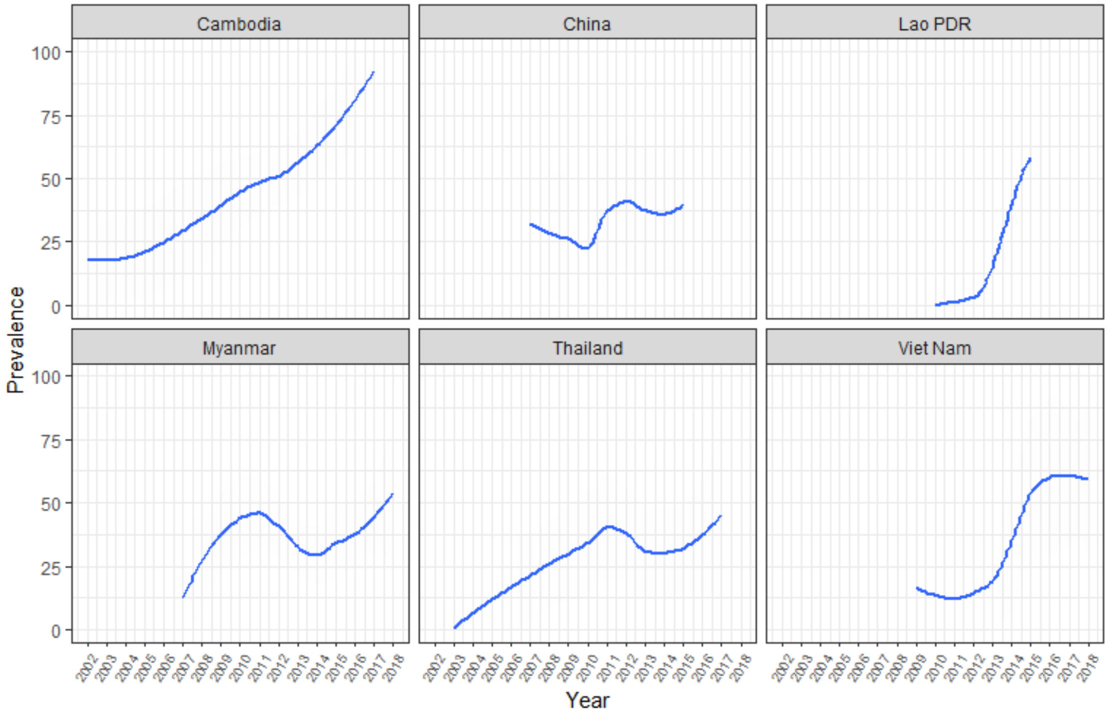

The overall prevalence of validated molecular markers by administrative unit per country over time from published k13 studies in the GMS. All countries show an increase in the 'validated' K13 markers.

Figure S5: WHO-validated markers in the GMS

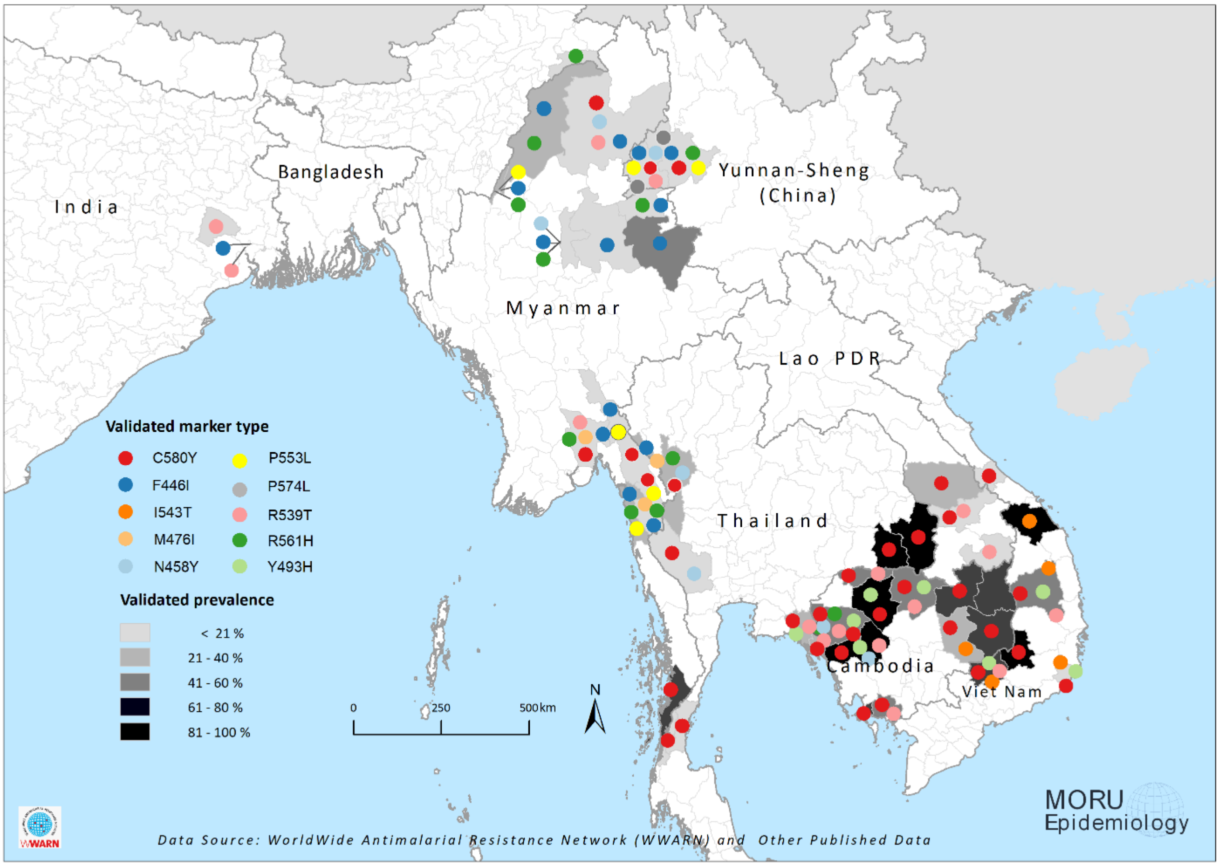

Distribution of the eight different validated SNPs and their prevalence. C580Y had wider distribution throughout the GMS particularly in the east. (Data was aggregated by administrative unit level one (Cambodia, Lao PDR, Thailand and Viet Nam and administrative level two for China, India and Myanmar)

Distribution of K13 markers in the GMS

Figure S6: Trend of WHO-Validated markers in selected locations in the GMS

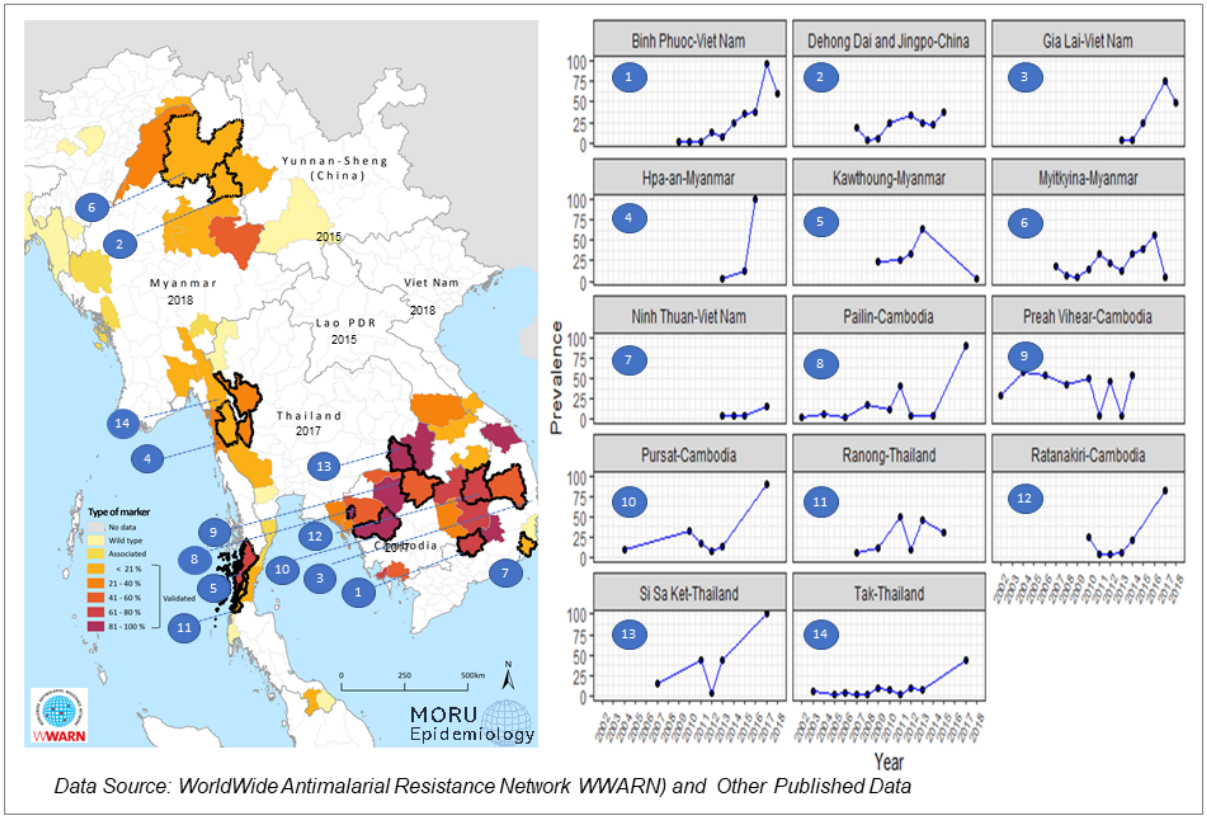

Prevalence of WHO-Validated markers over time for selected administrative subunits in the GMS with a minimum of 3 years of data (highlighted by black borders on the map and numbered in blue circles on the plots)

Figure S7. Temporal and spatial trends of K13 markers in the GMS (additional MS Power Point file)

This graphic interchange file displays the change of the overall prevalence of validated molecular markers by administrative unit over time from published k13 studies in the GMS. All countries show an increase in the 'validated' K13 markers.

Figure S8: Temporal trends of K13 marker prevalence in the GMS

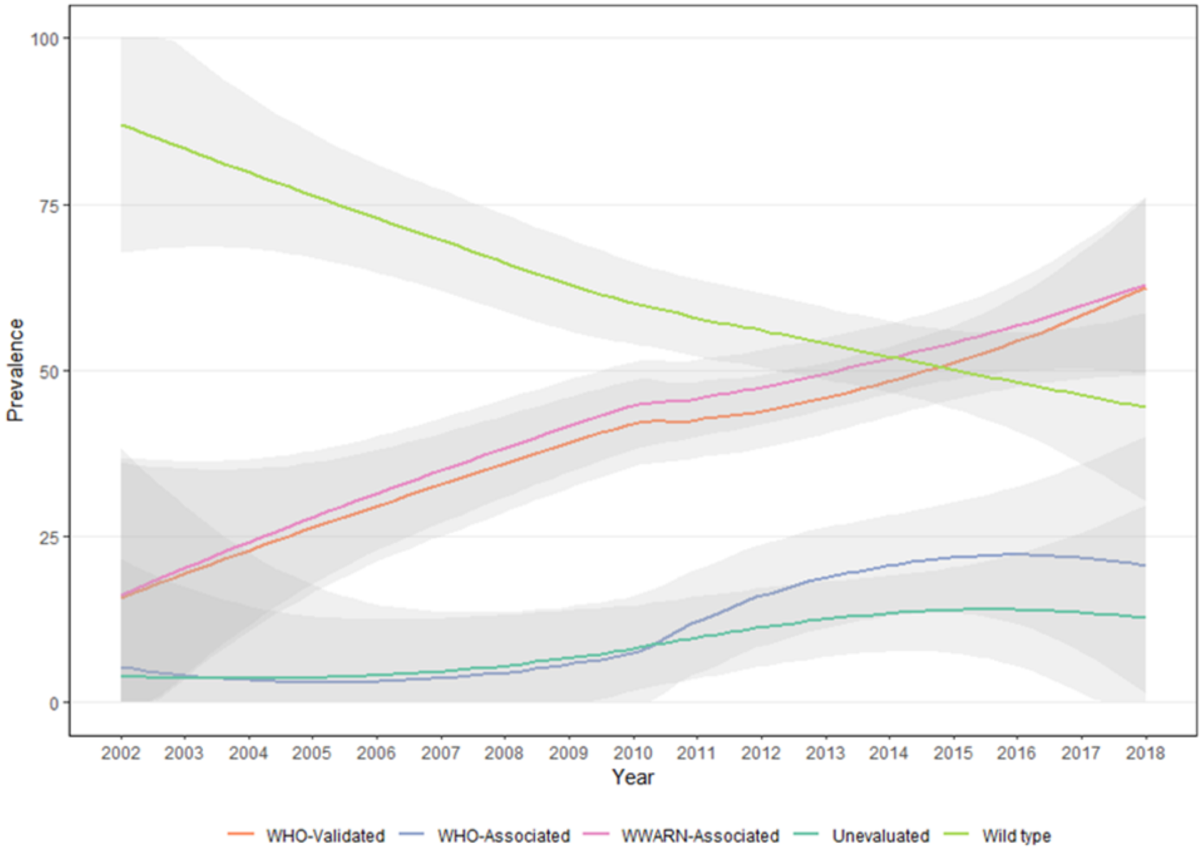

Annual prevalence of all K13 markers grouped by category. The lines represent the mean value and bands the 95% confidence intervals. The WHO validated, WHO and WWARN associated markers increased over time from 2002 to 2018 with a decrease in wild type parasites. However, there were wide confidence margins at both ends due to smaller sample sizes.

Figure S9: Distribution of K13 markers in South Asia

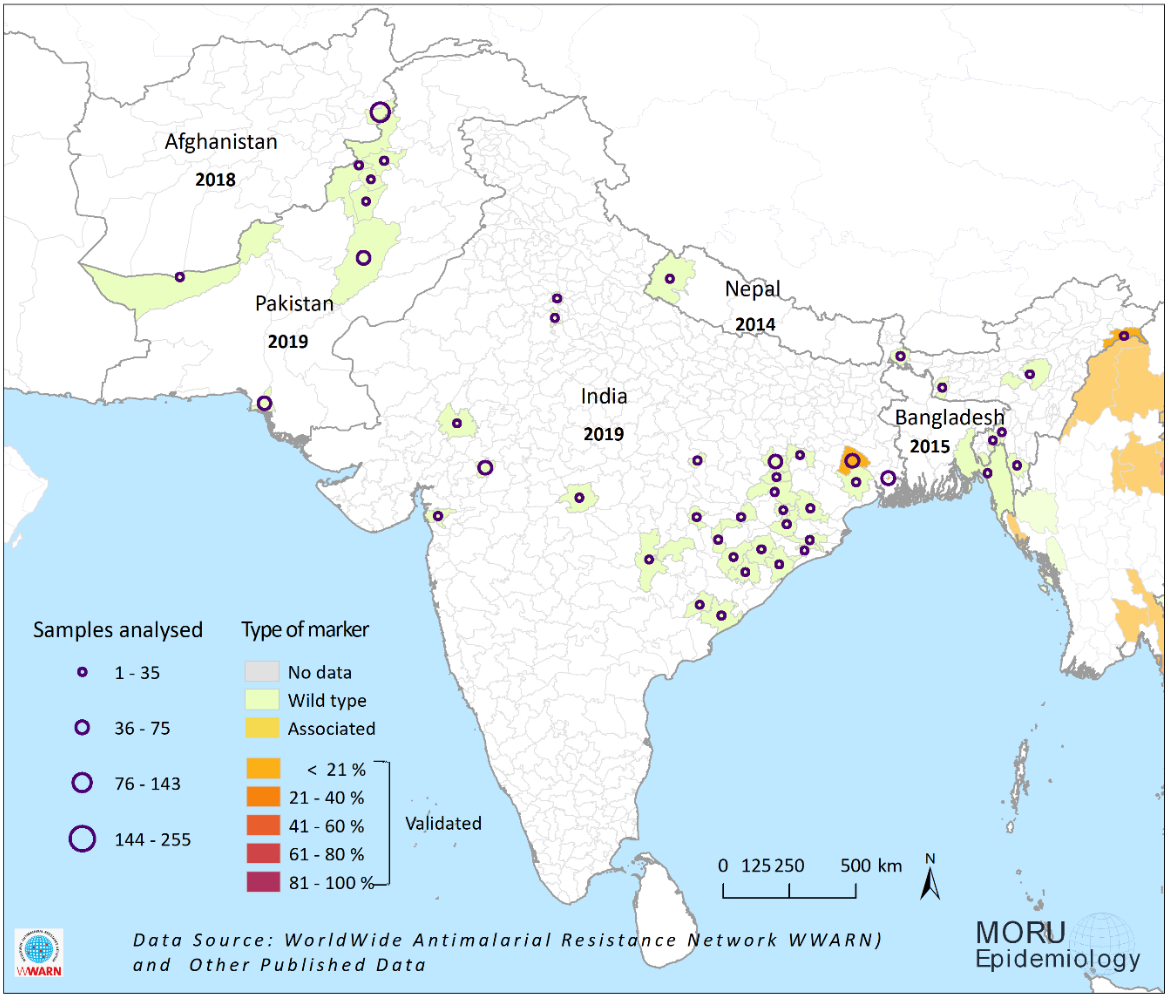

K13 markers in Afghanistan, Bangladesh, India, Nepal and Pakistan. Except for India which had WHO-validated K13 markers (F446I, R539T, R561H/C), other locations had only wild type parasites.

Table S5. K13 mutations by category.

Categories	SNPs
WHO validated/confirmed	C580Y, F446I, I543T, M476I, N458Y, P553L, P574L, R539T, R561H, Y493H
WHO associated	A481V, A675V, C469F, C469Y/F, E252Q, F673I, G449A, G449A/D, G538V, N537I, P441L, P527H, P574L, R515K, V568G
WWARN associated	A481V, A675V, C580Y, C580C/Y, E252Q, F446I, G449A, G449A/D, G538V, I543T, M476I, N458Y, N537I, P441L, P527H, P553L, P574L, P667T, R515K, R539T, R539R/T, R561H, R561H/C, V568G, Y493H
Unevaluated	A481T, A486V, A504T, A676D, C447Y, C469F, C469Y, C469Y/F, C580F, D281V, D452E, D464E, D512N, D516Y, D584V, E252K, E455K, E556D, E567D, E605G, E605K, E643K, F451I, F483S, F495L, F614L, F673I, G497V, G533A, G533S, G548S, G553A, H366L, H719N, I250T, I437T, I590T, I646L, K189T, K438N, K479I, K503N, L488M, L492S, L618L, M476I , M476V, M562I, M579T, M608K, M608V, N490T, N525D, N554S, N609S, N632D, N657H, N664S, N672R, P443Q, P443S, P553S, P667Q, P667R, P701R, R239Q, R513H, R528G, R528G/T, R529K, R575K, R575K/L, S459L, S485N, S549Y, S621F, S623C, S700L, T474I, T508N, T535A, T535M, T573A, V445G, V454I, V494I, V510G, V510M, V520I, V603E, V637A, V692L, Y493C, Y500C, Y511H, Y541H, Y604H
Not associated	A578S
Wild type	Wild type

A table showing various Single Nucleotide Polymorphisms (SNPs) and their categories as reported in the study.

Tool S1. The proposed K13 marker study reporting criteria (additional excel file).

To pool data, evaluate trends spatially and temporally, we hereby propose a tool that combines minimal essential information to be included when reporting K13 molecular markers. This tool consists of four excel sheets which include more information about the tool on sheet (P1-About), the tool to be completed (P2-Tool) horizontally for each sample, variables covered and their meaning (P3-Variables) and their further instructions (P4-Instructions). This tool can also be accessed on the WWARN Malaria Clinical Trials Toolkit (https://www.wwarn.org/tools-resources/kelch-markers-toolkit).

Table S6. Kelch 13 markers publications.

S.No	PMID	Title	Authors	Eligible/reason for not-eligible
1	28161569	The spread of artemisinin-resistant Plasmodium falciparum in the Greater Mekong subregion: a molecular epidemiology observational study. Lancet Infect Dis. 2017;17(5):491–7.	Imwong M, Suwannasin K, Kunasol C, Sutawong K, Mayxay M, Rekol H, et al.	eligible
2	27332904	A Worldwide Map of Plasmodium falciparum K13-Propeller Polymorphisms. N Engl J Med. 2016 Jun;374(25):2453–64	Ménard D, Khim N, Beghain J, Adegnika AA, Shafiul-Alam M, Amodu O, et al.	eligible
3	28137815	13 Propeller Mutations in Plasmodium falciparum Populations in Regions of Malaria Endemicity in Vietnam from 2009 to 2016. Antimicrob Agents Chemother. 2017 Apr;61(4).	Thuy-Nhien N, Tuyen NK, Tong NT, Vy NT, Thanh NV, Van HT, et al.	eligible
4	25180241	Independent Emergence of Artemisinin Resistance Mutations Among Plasmodium falciparum in Southeast Asia. J Infect Dis. 2015 Mar 1;211(5):670–9.	Takala-Harrison S, Jacob CG, Arze C, Cummings MP, Silva JC, Dondorp AM, et al.	eligible
5	25075834	Spread of artemisinin resistance in Plasmodium falciparum malaria. N Engl J Med. 2014 Jul;371(5):411–23.	Ashley EA, Dhorda M, Fairhurst RM, Amaratunga C, Lim P, Suon S, et al.	eligible
6	25224002	Delayed parasite clearance after treatment with dihydroartemisinin-piperaquine in Plasmodium falciparum malaria patients in central Vietnam. Antimicrob Agents Chemother. 2014 Dec;58(12):7049–55.	Thriemer K, Hong N Van, Rosanas-Urgell A, Phuc BQ, Ha DM, Pockele E, et al.	eligible
7	26616851	An outbreak of artemisinin resistant falciparum malaria in Eastern Thailand. Sci Rep. 2015 Nov 30;5:17412.	Imwong M, Jindakhad T, Kunasol C, Sutawong K, Vejakama P, Dondorp AM.	eligible

8	25836766	Selection and Spread of Artemisinin-Resistant Alleles in Thailand Prior to the Global Artemisinin Resistance Containment Campaign. PLoS Pathog. 2015 Apr;11(4):e1004789.	Talundzic E, Okoth SA, Congpuong K, Plucinski MM, Morton L, Goldman IF, et al.	eligible
9	27313266	Declining Efficacy of Artemisinin Combination Therapy Against P. Falciparum Malaria on the Thai-Myanmar Border (2003-2013): The Role of Parasite Genetic Factors. Clin Infect Dis an Off Publ Infect Dis Soc Am. 2016 Sep;63(6):784–91	Phyo AP, Ashley EA, Anderson TJC, Bozdech Z, Carrara VI, Sripraawat K, et al.	eligible
10	20689583	An open-label, randomised study of dihydroartemisinin-piperaquine versus artesunate-mefloquine for falciparum malaria in Asia. PLoS One. 2010 Jul;5(7):e11880.	Valecha N, Phyo AP, Mayxay M, Newton PN, Krudsood S, Keomany S, et al.	eligible
11	25704894	Spread of artemisinin-resistant Plasmodium falciparum in Myanmar: a cross-sectional survey of the K13 molecular marker. Lancet Infect Dis. 2015 Apr;15(4):415–21.	Tun KM, Imwong M, Lwin KM, Win AA, Hlaing TM, Hlaing T, et al.	eligible
12	27109419	Investigation and control of a Plasmodium falciparum malaria outbreak in Shan Special Region II of Myanmar along the China-Myanmar Border from June to December 2014. Infect Dis poverty. 2016 Apr;5:32.	Liu H, Xu J-W, Yang H-L, Li M, Sun C-D, Yin Y-J, et al.	eligible
13	25927592	Prevalence of K13-propeller polymorphisms in Plasmodium falciparum from China-Myanmar border in 2007-2012. Malar J. 2015 Apr;14:168.	Wang Z, Shrestha S, Li X, Miao J, Yuan L, Cabrera M, et al.	eligible
14	25537878	Molecular assessment of artemisinin resistance markers, polymorphisms in the k13 propeller, and a multidrug-resistance gene in the eastern and western border areas of Myanmar. Clin Infect Dis an Off	Nyunt MH, Hlaing T, Oo HW, Tin-Oo L-LK, Phway HP, Wang B, et al.	eligible

		Publ Infect Dis Soc Am. 2015 Apr;60(8):1208–15.		
15	27788228	Prevalence of Plasmodium falciparum Molecular Markers of Antimalarial Drug Resistance in a Residual Malaria Focus Area in Sabah, Malaysia. PLoS One. 2016;11(10):e0165515.	Norahmad NA, Mohd Abd Razak MR, Abdullah NR, Sastu UR, Imwong M, Muniandy PK, et al.	eligible
16	26688755	No Polymorphism in Plasmodium falciparum K13 Propeller Gene in Clinical Isolates from Kolkata, India. J Pathog. 2015;2015:374354.	Chatterjee M, Ganguly S, Saha P, Bankura B, Basu N, Das M, et al	eligible
17	27737665	Therapeutic efficacy of artemether-lumefantrine for the treatment of uncomplicated Plasmodium falciparum malaria from three highly malarious states in India. Malar J. 2016 Oct;15(1):498.	Bharti PK, Shukla MM, Ringwald P, Krishna S, Singh PP, Yadav A, et al.	eligible
18	25691626	Surveillance of artemisinin resistance in Plasmodium falciparum in India using the kelch13 molecular marker. Antimicrob Agents Chemother. 2015 May 1;59(5):2548–53.	Mishra N, Prajapati SK, Kaitholia K, Bharti RS, Srivastava B, Phookan S, et al.	eligible
19	25691632	Amplification of pfmdr1, pfcrt, pvmdr1, and K13 propeller polymorphisms associated with Plasmodium falciparum and Plasmodium vivax isolates from the China-Myanmar border. Antimicrob Agents Chemother. 2015 May 1;59(5):2554–9.	Feng J, Zhou D, Lin Y, Xiao H, Yan H, Xia Z.	eligible
20	25910630	A Single Mutation in K13 Predominates in Southern China and Is Associated With Delayed Clearance of Plasmodium falciparum Following Artemisinin Treatment. J Infect Dis. 2015 Nov 15;212(10):1629–35.	Huang F, Takala-Harrison S, Jacob CG, Liu H, Sun X, Yang H, et al.	eligible
21	26695060	Plasmodium falciparum dihydroartemisinin-piperaquine failures in Cambodia are associated with mutant K13 parasites presenting high	Duru V, Khim N, Leang R, Kim S, Domergue A, Kloeung N, et al.	eligible

		survival rates in novel piperaquine in vitro assays: retrospective and prospective investigations. BMC Med. 2015 Dec;13:305.		
22	26774243	Dihydroartemisinin-piperaquine resistance in Plasmodium falciparum malaria in Cambodia: a multisite prospective cohort study. Lancet Infect Dis. 2016 Mar;16(3):357–65.	Amaratunga C, Lim P, Suon S, Sreng S, Mao S, Sopha C, et al.	eligible
23	24352242	A molecular marker of artemisinin-resistant Plasmodium falciparum malaria. Nature. 2014 Jan;505(7481):50–5.	Ariey F, Witkowski B, Amaratunga C, Beghain J, Langlois A-C, Khim N, et al.	eligible
24	25288380	Plasmodium prevalence and artemisinin-resistant falciparum malaria in Preah Vihear Province, Cambodia: a cross-sectional population-based study. Malar J. 2014 Oct;13:394.	Bosman P, Stassijns J, Nackers F, Canier L, Kim N, Khim S, et al.	eligible
25	25877962	Dihydroartemisinin-piperaquine failure associated with a triple mutant including kelch13 C580Y in Cambodia: an observational cohort study. Lancet Infect Dis. 2015 Jun;15(6):683–91.	Spring MD, Lin JT, Manning JE, Vanachayangkul P, Somethy S, Bun R, et al.	eligible
26	25404021	Mutations in Plasmodium falciparum K13 propeller gene from Bangladesh (2009-2013). Malar J. 2014 Nov;13:431.	Mohon AN, Alam MS, Bayih AG, Folefoc A, Shahinas D, Haque R, et al.	eligible
27	26917051	Clinical trials of artesunate plus sulfadoxine-pyrimethamine for Plasmodium falciparum malaria in Afghanistan: maintained efficacy a decade after introduction. Malar J. 2016 Feb;15:121.	Awab GR, Imwong M, Pukrittayakamee S, Alim F, Hanpithakpong W, Tarning J, et al.	eligible
28	30535043	Novel pfkelch13 Gene Polymorphism Associates With Artemisinin Resistance in Eastern India. Clin Infect Dis an Off Publ Infect Dis Soc Am. 2019 Sep;69(7):1144–52.	Das S, Manna S, Saha B, Hati AK, Roy S.	eligible

29	29793059	Longitudinal surveillance of drug resistance in Plasmodium falciparum isolates from the China-Myanmar border reveals persistent circulation of multidrug resistant parasites. Int J Parasitol Drugs drug Resist. 2018 Aug;8(2):320–8.	Bai Y, Zhang J, Geng J, Xu S, Deng S, Zeng W, et al.	eligible
30	27036739	Parasite clearance rates in Upper Myanmar indicate a distinctive artemisinin resistance phenotype: a therapeutic efficacy study. Malar J. 2016 Mar;15:185.	Tun KM, Jeeyapant A, Imwong M, Thein M, Aung SSM, Hlaing TM, et al.	eligible
31	29345221	Therapeutic Response to Dihydroartemisinin-Piperaquine for P. falciparum and P. vivax Nine Years after Its Introduction in Southern Papua, Indonesia. Am J Trop Med Hyg. 2018 Mar;98(3):677–82.	Poespoprodjo JR, Kenangalem E, Wafom J, Chandrawati F, Puspitasari AM, Ley B, et al.	eligible
32	29813085	Resistance screening and trend analysis of imported falciparum malaria in NSW, Australia (2010 to 2016).	Prosser PC, Meyer MW, Ellis EJ, Lee LR	eligible
33	27301553	Plasmodium falciparum parasite population structure and gene flow associated to antimalarial drugs resistance in Cambodia. Malar J. 2016 Jun;15:319.	Dwivedi A, Khim N, Reynes C, Ravel P, Ma L, Tichit M, et al.	eligible
34	27234446	Asymptomatic Plasmodium infections in 18 villages of southern Savannakhet Province, Lao PDR (Laos). Malar J. 2016 May;15(1):296.	Phommasone K, Adhikari B, Henriques G, Pongvongsa T, Phongmany P, von Seidlein L, et al.	eligible
35	28806957	Clinical and molecular surveillance of artemisinin resistant falciparum malaria in Myanmar (2009-2013). Malar J. 2017;16(1):333.	Nyunt MH, Soe MT, Myint HW, Oo HW, Aye MM, Han SS, et al.	eligible
36	30939179	Clinical impact of the two ART resistance markers, K13 gene mutations and DPC3 in Vietnam. PLoS One. 2019;14(4).	Pau MC, Pantaleo A, Tsamesidis I, Hoang H, Tran AT, Nguyen TLH, et al.	eligible
37	28086775	Rapid decline in the susceptibility of Plasmodium	Thanh NV, Thuy-Nhien N, Tuyen NTK, Tong	eligible

		falciparum to dihydroartemisinin-piperaquine in the south of Vietnam. Malar J. 2017 Jan;16(1):27.	NT, Nha-Ca NT, Dong LT, et al.	
38	31345710	Determinants of dihydroartemisinin-piperaquine treatment failure in Plasmodium falciparum malaria in Cambodia, Thailand, and Vietnam: a prospective clinical, pharmacological, and genetic study. Lancet Infect Dis. 2019 Sep;19(9):952–61.	van der Pluijm RW, Imwong M, Chau NH, Hoa NT, Thuy-Nhien NT, Thanh NV, et al.	eligible
39	27585957	Assessing the asymptomatic reservoir and dihydroartemisinin-piperaquine effectiveness in a low transmission setting threatened by artemisinin resistant Plasmodium falciparum. Malar J. 2016 Sep;15(1):446.	Falq G, Van Den Bergh R, De Smet M, Etienne W, Nguon C, Rekol H, et al.	eligible
40	28494763	Artemisinin resistance without pfkelch13 mutations in Plasmodium falciparum isolates from Cambodia. Malar J. 2017 Dec 12;16(1):195.	Mukherjee A, Bopp S, Magistrado P, Wong W, Daniels R, Demas A, et al.	eligible
41	29334942	Poor response to artesunate treatment in two patients with severe malaria on the Thai–Myanmar border. Malar J. 2018 Dec 15;17(1):30.	Phyo AP, Win KK, Thu AM, Swe LL, Htike H, Beau C, et al.	eligible
42	27234587	Sustained efficacy of artesunate-sulfadoxine-pyrimethamine against Plasmodium falciparum in Yemen and a renewed call for an adjunct single dose primaquine to clear gametocytes. Malar J. 2016 May;15(1):295.	Atroosh WM, Al-Mekhlafi HM, Snounou G, Al-Jasari A, Sady H, Nasr NA, et al.	eligible
43	28388902	Therapeutic efficacy and artemisinin resistance in northern Myanmar: evidence from in vivo and molecular marker studies. Malar J. 2017;16(1):143.	Myint MK, Rasmussen C, Thi A, Bustos D, Ringwald P, Lin K.	eligible

44	27343362	Molecular markers associated with resistance to commonly used antimalarial drugs among Plasmodium falciparum isolates from a malaria-endemic area in Taiz governorate-Yemen during the transmission season. Acta Trop. 2016 Oct;162:174–9.	Alareqi LMQ, Mahdy MAK, Lau Y-L, Fong M-Y, Abdul-Ghani R, Mahmud R.	eligible
45	28249583	Molecular surveillance of artemisinin resistance falciparum malaria among migrant goldmine workers in Myanmar. Malar J. 2017 Dec 1;16(1):97.	Nyunt MH, Wang B, Aye KM, Aye KH, Han J-H, Lee S-K, et al.	eligible
46	27084511	Examining Plasmodium falciparum and P. vivax clearance subsequent to antimalarial drug treatment in the Myanmar-China border area based on quantitative real-time polymerase chain reaction. BMC Infect Dis. 2016 Apr;16:154.	Lo E, Nguyen J, Oo W, Hemming-Schroeder E, Zhou G, Yang Z, et al.	eligible
47	26548510	Natural selection of K13 mutants of Plasmodium falciparum in response to artemisinin combination therapies in Thailand. Clin Microbiol Infect. 2016 Mar;22(3):285.e1-285.e8.	Putaporntip C, Kuamsab N, Kosuwin R, Tantiwattanasub W, Vejakama P, Sueblinvong T, et al.	eligible
48	29996844	Effectiveness and safety of 3 and 5 day courses of artemether-lumefantrine for the treatment of uncomplicated falciparum malaria in an area of emerging artemisinin resistance in Myanmar. Malar J. 2018 Jul;17(1):258.	Tun KM, Jeeyapant A, Myint AH, Kyaw ZT, Dhorda M, Mukaka M, et al.	eligible
49	28806961	Molecular analysis demonstrates high prevalence of chloroquine resistance but no evidence of artemisinin resistance in Plasmodium falciparum in the Chittagong Hill Tracts of Bangladesh. Malar J. 2017;16(1):335.	Alam MS, Ley B, Nima MK, Johora FT, Hossain ME, Thriemer K, et al.	eligible

50	31009824	In vitro susceptibility of Plasmodium falciparum isolates from the China-Myanmar border area to artemisinins and correlation with K13 mutations. Int J Parasitol Drugs drug Resist. 2019 Apr 10;10:20–7.	Zhang J, Li N, Siddiqui FA, Xu S, Geng J, Zhang J, et al.	eligible
51	METFGENRE	METF-SMRU/GenRe	Prof. Francois Nosten and GenRe-Mekong Project	eligible
52	30883571	Artemisinin resistance-associated markers in Plasmodium falciparum parasites from the China-Myanmar border: predicted structural stability of K13 propeller variants detected in a low-prevalence area. Carvalho LH, editor. PLoS One. 2019 Mar 18;14(3):e0213686.	He Y, Campino S, Diez Benavente E, Warhurst DC, Beshir KB, Lubis I, et al.	eligible
53	31239407	K13 propeller domain mutations and pfmdr1 amplification in isolates of Plasmodium falciparum collected from Thai-Myanmar border area in 2006-2010. Folia Parasitol (Praha). 2019 May;66.	Phompradit P, Chaijaroenkul W, Muhamad P, Na-Bangchang K.	eligible
54	31251812	Pyronaridine-artesunate Efficacy and Safety in Uncomplicated Plasmodium falciparum Malaria in Areas of Artemisinin-resistant Falciparum in Viet Nam (2017-2018). Clin Infect Dis an Off Publ Infect Dis Soc Am. 2020 May;70(10):2187–95.	Quang Bui P, Hong Huynh Q, Thanh Tran D, Thanh Le D, Quang Nguyen T, Van Truong H, et al.	eligible
55	28322709	Treatment Failure of Dihydroartemisinin/Piperaquine for Plasmodium falciparum Malaria, Vietnam. Emerg Infect Dis. 2017;23(4):715.	Phuc BQ, Rasmussen C, Duong TT, Dong LT, Loi MA, Ménard D, et al.	eligible
56	29110709	The prevalence, incidence and prevention of Plasmodium falciparum infections in forest rangers in Bu Gia Map National Park, Binh Phuoc province,	Son DH, Thuy-Nhien N, von Seidlein L, Le Phuc-Nhi T, Phu NT, Tuyen NTK, et al.	eligible

		Vietnam: a pilot study. Malar J. 2017 Dec 6;16(1):444.		
57	29178921	Plasmodium falciparum Kelch 13 mutations and treatment response in patients in Hpa-Pun District, Northern Kayin State, Myanmar. Malar J. 2017 Dec 25;16(1):480.	Bonnington CA, Phyo AP, Ashley EA, Imwong M, Sriprawat K, Parker DM, et al.	eligible
58	28549390	Clinical and molecular monitoring of Plasmodium falciparum resistance to antimalarial drug (artesunate+sulphadoxine-pyrimethamine) in two highly malarious district of Madhya Pradesh, Central India from 2012-2014. Pathog Glob Health. 2017 Jun;111(4):186–94.	Mishra S, Bharti PK, Shukla MM, Ali NA, Kashyotia SS, Kumar A, et al.	eligible
59	28903755	Prevalence of K13 mutation and Day-3 positive parasitaemia in artemisinin-resistant malaria endemic area of Cambodia: a cross-sectional study. Malar J. 2017 Sep;16(1):372.	Kheang ST, Sovannaroth S, Ek S, Chy S, Chhun P, Mao S, et al.	eligible
60	31833468	Artemether-lumefantrine and dihydroartemisinin-piperaquine retain high efficacy for treatment of uncomplicated plasmodium falciparum malaria in Myanmar. Am J Trop Med Hyg. 2020;102(3):598–604.	Han KT, Lin K, Myint MK, Thi A, Aye KH, Han ZY, et al.	eligible
61	31791329	Genetic profiling of the Plasmodium falciparum parasite population in uncomplicated malaria from India. Malar J. 2019 Dec 2;18(1):385.	Kumar A, Singh SP, Bhatt R, Singh V.	eligible
62	31548652	Characterisation of drug resistance and genetic diversity of Plasmodium falciparum parasites from Tripura, Northeast India. Sci Rep. 2019 Dec 1;9(1).	Patgiri SJ, Sarma K, Sarmah N, Bhattacharyya N, Sarma DK, Nirmolia T, et al.	eligible
63	31533403	Molecular surveillance of Pfkelch13 and Pfmdr1 mutations in Plasmodium falciparum isolates from	Khammanee T, Sawangjaroen N, Buncherd H, Tun AW, Thanapongpichat S.	eligible

		southern Thailand. Korean J Parasitol. 2019 Aug 1;57(4):369–77.		
64	31416468	Molecular detection of drug resistant malaria in Southern Thailand. Malar J. 2019 Aug;18(1):275.	Noisang C, Prosser C, Meyer W, Chemoh W, Ellis J, Sawangjaroen N, et al.	eligible
65	31069209	Investigation and evaluation of genetic diversity of kelch 13 polymorphisms in plasmodium falciparum from southern China. Front Public Heal. 2019;7(APR).	Feng J, Kong X, Xu D, Yan H, Zhou H, Tu H, et al.	eligible
66	32524960	Efficacy and Safety of Pyronaridine-Artesunate for the Treatment of Uncomplicated Plasmodium falciparum and Plasmodium vivax Malaria in Myanmar. Am J Trop Med Hyg. 2020 Sep;103(3):1088–93.	Han KT, Lin K, Han ZY, Myint MK, Aye KH, Thi A, et al.	eligible
67	32258150	Sequence Analysis of the K13-Propeller Gene in Artemisinin Challenging Plasmodium falciparum Isolates from Malaria Endemic Areas of Odisha, India: A Molecular Surveillance Study. Biomed Res Int. 2020;2020:8475246.	Rana R, Ranjit M, Bal M, Khuntia HK, Pati S, Krishna S, et al.	eligible
68	32513171	Surveillance of genetic markers associated with Plasmodium falciparum resistance to artemisinin-based combination therapy in Pakistan, 2018-2019. Malar J. 2020 Jun 8;19(1):206.	Khan AQ, Khan AQ, Pernaute-Lau L, Pernaute-Lau L, Khattak AA, Luijcx S, et al.	eligible
69	33320907	Emergence of artemisinin-resistant Plasmodium falciparum with kelch13 C580Y mutations on the island of New Guinea. PLoS Pathog. 2020 Dec 15;16(12)	Miotto O, Sekihara M, Tachibana SI, Yamauchi M, Pearson RD, Amato R, et al.	eligible
70	33168025	Kelch 13-propeller polymorphisms in Plasmodium falciparum from Jazan region, southwest Saudi Arabia. Malar J. 2020 Dec 1;19(1):397.	Dafalla OM, Alzahrani M, Sahli A, Al Helal MA, Alhazmi MM, Noureldin EM, et al.	eligible

71	32854686	Efficacy of artemether-lumefantrine for treating uncomplicated Plasmodium falciparum cases and molecular surveillance of drug resistance genes in Western Myanmar. Malar J. 2020 Aug 27;19(1)	Wu Y, Soe MT, Aung PL, Zhao L, Zeng W, Menezes L, et al.	eligible
72	31351071	In vitro synergistic interaction of potent 4-aminoquinolines in combination with dihydroartemisinin against chloroquine-resistant Plasmodium falciparum. Acta Trop. 2019 Nov;199:105109.	Agarwal D, Singh S, Gupta RD, Awasthi SK.	antimalarial-yes, K13 information-no, clinical study/invivo study-yes, Asia-yes, other-no
73	31345709	Evolution and expansion of multidrug-resistant malaria in southeast Asia: a genomic epidemiology study. Lancet Infect Dis. 2019 Sep;19(9):943–51.	Hamilton WL, Amato R, van der Pluijm RW, Jacob CG, Quang HH, Thuy-Nhien NT, et al.	antimalarial-yes, K13 information-no, clinical study/invivo study-yes, Asia-yes, other-used previously published samples
74	31267499	An Update on Artemisinin Resistance. Methods Mol Biol. 2019; 2013:141–9.	Ariey F, Ménard D.	antimalarial-yes, K13 information-no, clinical study/invivo study-yes, Asia-yes, other-review
75	31232939	Intervention of artemisinin in macular edema associated with retinal vein occlusion: A protocol for a systematic review and meta-analysis. Medicine (Baltimore). 2019 Jun;98(25):e16044.	Xu J, Hao X, Lu B, Ming J, Li X, Qi Y, et al.	antimalarial-yes, K13 information-no, clinical study/invivo study-no, Asia-no, other-protocol
76	31210357	Artemether for severe malaria. Cochrane database Syst Rev. 2019 Jun;6(6):CD010678.	Esu EB, Effa EE, Opie ON, Meremikwu MM.	antimalarial-yes, K13 information-no, clinical study/invivo study-yes, Asia-yes, other-review
77	31185976	An improved nucleic acid extraction method from dried blood spots for amplification of Plasmodium falciparum kelch13 for detection of artemisinin resistance. Malar J. 2019 Jun;18(1):192.	Zainabadi K, Nyunt MM, Plowe C V.	antimalarial-yes, K13 information-yes, clinical study/invivo study-no, Asia-no, other-no
78	31108084	Modulation of in vitro antimalarial responses by polymorphisms in Plasmodium	Gendrot M, Wague Gueye M, Tsombeng Foguim F, Madamet M,	antimalarial-no, K13 information-no, clinical study/invivo

		falciparum ABC transporters (pfmdr1 and pfmdr5). Acta Trop. 2019 Aug; 196:126–34.	Wade KA, Bou Kounta M, et al.	study-no, Asia-no, other-no
79	31075171	Antimalarial Immunity to Measures of Parasite Clearance in Therapeutic Efficacy Studies of Artemisinin Derivatives. J Infect Dis. 2019 Aug;220(7):1178–87.	O'Flaherty K, Ataíde R, Zaloumis SG, Ashley EA, Powell R, Feng G, et al.	antimalarial-no, K13 information-no, clinical study/invivo study-no, Asia-no, other-no
80	31015034	Efficacy and resistance of different artemisinin-based combination therapies: a systematic review and network meta-analysis. Parasitol Int. 2020 Feb;74:101919.	Mathenge PG, Low SK, Vuong NL, Mohamed MYF, Faraj HA, Alieldin GI, et al.	antimalarial-yes, K13 information-no, clinical study/invivo study-no, Asia-yes, other-review
81	30995310	Geographic expansion of artemisinin resistance. J Travel Med. 2019 Jun;26(4).	Müller O, Lu GY, von Seidlein L.	antimalarial-yes, K13 information-yes, clinical study/invivo study-no, Asia-yes, other-review
82	30768615	The impact of targeted malaria elimination with mass drug administrations on falciparum malaria in Southeast Asia: A cluster randomised trial. PLoS Med. 2019 Feb;16(2):e1002745.	von Seidlein L, Peto TJ, Landier J, Nguyen T-N, Tripura R, Phommasone K, et al.	antimalarial-yes, K13 information-yes, clinical study/invivo study-yes, Asia-yes, other-no
83	30753425	Targeting malaria parasite invasion of red blood cells as an antimalarial strategy. FEMS Microbiol Rev. 2019 May;43(3):223–38.	Burns AL, Dans MG, Balbin JM, de Koning-Ward TF, Gilson PR, Beeson JG, et al.	antimalarial-yes, K13 information-no, clinical study/invivo study-no, Asia-yes, other-no
84	30717149	Artemisinin Combination Therapies (ACTs): Do Not Forget the Partner Drug! Trop Med Infect Dis. 2019 Feb;4(1).	Nsanzabana C.	antimalarial-yes, K13 information-yes, clinical study/invivo study-no, Asia-yes, other-no
85	30701807	Problems of clinical diagnosis and treatment of P. falciparum malaria in Russian Federation. Vol. 90, Terapevticheskii arkhiv. Russia (Federation); 2018. p. 4–8.	Sergiev VP, Baranova AM, Kozhevnikova GM, Tokmalayev AK, Chernyshov D V, Chentsov VB, et al.	antimalarial-yes, K13 information-no, clinical study/invivo study-yes, Asia-no, other-no
86	30654808	Susceptibility of Plasmodium falciparum to artemisinins and Plasmodium vivax to chloroquine in Phuoc Chien Commune, Ninh Thuan	Phong NC, Chavchich M, Quang HH, San NN, Birrell GW, Chuang I, et al.	antimalarial-yes, K13 information-no, clinical study/invivo study-yes, Asia-yes, other-no

		Province, south-central Vietnam. Malar J. 2019 Jan;18(1):10.		
87	30620055	Pyronaridine-artesunate for treating uncomplicated Plasmodium falciparum malaria. Cochrane database Syst Rev. 2019 Jan;1(1):CD006404.	Pryce J, Hine P.	antimalarial-yes, K13 information-no, clinical study/invivo study-no, Asia-yes, other-review
88	30607137	Patients' adherence to artemisinin-based combination therapy and healthcare workers' perception and practice in Savannakhet province, Lao PDR. Trop Med Health. 2018;46:44.	Takahashi E, Nonaka D, Iwagami M, Phoutnalong V, Chanthakoumane K, Kobayashi J, et al.	antimalarial-yes, K13 information-no, clinical study/invivo study-no, Asia-no, other-no
89	30580023	Overexpression of plasmepsin II and plasmepsin III does not directly cause reduction in Plasmodium falciparum sensitivity to artesunate, chloroquine and piperaquine. Int J Parasitol Drugs drug Resist. 2019 Apr;9:16–22.	Loesbanluechai D, Kotanan N, de Cozar C, Kochakarn T, Ansbro MR, Chotivanich K, et al.	antimalarial-yes, K13 information-yes, clinical study/invivo study-yes, Asia-yes, other-no
90	30572592	Artemether and Praziquantel: Origin, Mode of Action, Impact, and Suggested Application for Effective Control of Human Schistosomiasis. Trop Med Infect Dis. 2018 Dec;3(4).	Bergquist R, Elmorshedy H.	antimalarial-yes, K13 information-no, clinical study/invivo study-no, Asia-no, other-no
91	30563521	Genetic association between the Pfk13 gene mutation and artemisinin resistance phenotype in Plasmodium falciparum isolates from Yunnan Province, China. Malar J. 2018 Dec;17(1):478.	Dong Y, Wang J, Sun A, Deng Y, Chen M, Xu Y, et al.	antimalarial-yes, K13 information-yes, clinical study/invivo study-no, Asia-yes, other-secondary data from another study already included
92	30558597	Origins and spread of novel genetic variants of sulfadoxine-pyrimethamine resistance in Plasmodium falciparum isolates in Indonesia. Malar J. 2018 Dec;17(1):475.	Basuki S, Fitriah, Risamasu PM, Kasmijati, Ariami P, Riyanto S, et al.	antimalarial-yes, K13 information-no, clinical study/invivo study-yes, Asia-yes, other-no
93	30514877	The origins of malaria artemisinin resistance defined by a genetic and transcriptomic	Zhu L, Tripathi J, Rocamora FM, Miotto O, van der Pluijm R, Voss TS, et al.	antimalarial-yes, K13 information-yes, clinical study/invivo study-no, Asia-yes,

		background. Nat Commun. 2018 Dec;9(1):5158.		other-secondary data from another study already included
94	30499404	Antimalarials: Review of Plasmepsins as Drug Targets and HIV Protease Inhibitors Interactions. Curr Top Med Chem. 2019;18(23):2022–8.	Miller Iii WA, Teye J, Achieng AO, Mogire RM, Akala H, Ong'echa JM, et al.	antimalarial-yes, K13 information-no, clinical study/invivo study-no, Asia-yes, other-review
95	30486796	Altered expression of K13 disrupts DNA replication and repair in Plasmodium falciparum. BMC Genomics. 2018 Nov;19(1):849.	Gibbons J, Button-Simons KA, Adapa SR, Li S, Pietsch M, Zhang M, et al.	antimalarial-yes, K13 information-yes, clinical study/invivo study-no, Asia-yes, other-transcriptonic lab study
96	30478733	Current scenario and future strategies to fight artemisinin resistance. Parasitol Res. 2019 Jan;118(1):29–42.	Pasupureddy R, Atul, Seshadri S, Pande V, Dixit R, Pandey KC.	antimalarial-yes, K13 information-yes, clinical study/invivo study-no, Asia-yes, other-review
97	30447701	K13-propeller gene polymorphisms in Plasmodium falciparum parasite population: a systematic review protocol of burden and associated factors. Syst Rev. 2018 Nov;7(1):199.	Ocan M, Akena D, Nsobya S, Kamya MR, Senono R, Kinengyere AA, et al.	antimalarial-yes, K13 information-yes, clinical study/invivo study-no, Asia-yes, other-review
98	30390647	The dynamic of asymptomatic Plasmodium falciparum infections following mass drug administrations with dihydroarteminisin-piperaquine plus a single low dose of primaquine in Savannakhet Province, Laos. Malar J. 2018 Nov;17(1):405.	Pongvongsa T, Phommasone K, Adhikari B, Henriques G, Chotivanich K, Hanboonkunupakarn B, et al.	antimalarial-yes, K13 information-no, clinical study/invivo study-yes, Asia-yes, other-did not asses kelch markers
99	30367653	A single nucleotide polymorphism in the Plasmodium falciparum atg18 gene associates with artemisinin resistance and confers enhanced parasite survival under nutrient deprivation. Malar J. 2018 Oct;17(1):391.	Breglio KF, Amato R, Eastman R, Lim P, Sa JM, Guha R, et al.	antimalarial-yes, K13 information-no, clinical study/invivo study-no, Asia-yes, other-no
100	30269689	High-level artemisinin-resistance with quinine co-resistance emerges in P. falciparum malaria under in	Tyagi RK, Gleeson PJ, Arnold L, Tahar R, Prieur E, Decosterd L, et al.	antimalarial-yes, K13 information-yes, clinical study/invivo

		vivo artesunate pressure. BMC Med. 2018 Oct;16(1):181.		study-no, Asia-yes, other-no
101	30154519	Surveillance of Antimalarial Resistance Pfcrt, Pfmdr1, and Pfkelch13 Polymorphisms in African Plasmodium falciparum imported to Shandong Province, China. Sci Rep. 2018 Aug 28;8(1):12951.	Xu C, Wei Q, Yin K, Sun H, Li J, Xiao T, et al.	antimalarial-yes, K13 information-yes, clinical study/invivo study-yes, Asia-yes, other-imported cases - travellers from African countries
102	30139985	Expression of key genes affecting artemisinin content in five Artemisia species. Sci Rep. 2018 Aug;8(1):12659.	Salehi M, Karimzadeh G, Naghavi MR, Naghdi Badi H, Rashidi Monfared S.	antimalarial-yes, K13 information-no, clinical study/invivo study-no, Asia-no, other-no
103	30135481	Fitness Loss under Amino Acid Starvation in Artemisinin-Resistant Plasmodium falciparum Isolates from Cambodia. Sci Rep. 2018 Aug;8(1):12622.	Bunditvorapoom D, Kochakarn T, Kotanan N, Modchang C, Kümpornsin K, Loesbanluechai D, et al.	antimalarial-yes, K13 information-no, clinical study/invivo study-yes, Asia-yes, other-no
104	30115924	Emerging Southeast Asian PfCRT mutations confer Plasmodium falciparum resistance to the first-line antimalarial piperaquine. Nat Commun. 2018 Aug;9(1):3314.	Ross LS, Dhingra SK, Mok S, Yeo T, Wicht KJ, Kümpornsin K, et al.	antimalarial-yes, K13 information-no, clinical study/invivo study-yes, Asia-yes, other-no
105	30071877	Implications of population-level immunity for the emergence of artemisinin-resistant malaria: a mathematical model. Malar J. 2018 Aug;17(1):279.	Scott N, Ataide R, Wilson DP, Hellard M, Price RN, Simpson JA, et al.	antimalarial-yes, K13 information-yes, clinical study/invivo study-no, Asia-yes, other-modelling study
106	30016713	Variation in intronic microsatellites and exon 2 of the Plasmodium falciparum chloroquine resistance transporter gene during modification of artemisinin combination therapy in Thailand. Infect Genet Evol J Mol Epidemiol Evol Genet Infect Dis. 2018 Nov;65:35–42.	Seethamchai S, Buppan P, Kuamsab N, Teeranaipong P, Putaporntip C, Jongwutiwes S.	antimalarial-yes, K13 information-no, clinical study/invivo study-no, Asia-yes, other-no
107	29980936	Dihydroartemisinin-piperaquine treatment failure in uncomplicated Plasmodium falciparum malaria case imported from Ethiopia.	Russo G, L'Episcopia M, Menegon M, Souza SS, Dongho BGD, Vullo V, et al.	antimalarial-yes, K13 information-no, clinical study/invivo study-yes, Asia-no, other-no

		Infection. 2018 Dec;46(6):867–70.		
108	29976207	Introduction of F446I mutation in the K13 propeller gene leads to increased ring survival rates in Plasmodium falciparum isolates. Malar J. 2018 Jul;17(1):248.	Wang J, Huang Y, Zhao Y, Ye R, Zhang D, Pan W.	antimalarial-yes, K13 information-yes, clinical study/invivo study-no, Asia-yes, other-transgenic study
109	29973212	Efficacy of two artemisinin-based combinations for the treatment of malaria in pregnancy in India: a randomized controlled trial. Malar J. 2018 Jul;17(1):246.	Anvikar AR, Kuepfer I, Mishra V, Bruce J, Arya T, Mishra DR, et al.	antimalarial-yes, K13 information-no, clinical study/invivo study-yes, Asia-yes, other-no evaluation of kelch markers
110	29843734	An innovative diagnostic technology for the codon mutation C580Y in kelch13 of Plasmodium falciparum with MinION nanopore sequencer. Malar J. 2018 May;17(1):217.	Imai K, Tarumoto N, Runtuwene LR, Sakai J, Hayashida K, Eshita Y, et al.	antimalarial-yes, K13 information-yes, clinical study/invivo study-no, Asia-yes, other-diagnostic study
111	29798745	Therapeutic and Transmission-Blocking Efficacy of Dihydroartemisinin/Piperaquine and Chloroquine against Plasmodium vivax Malaria, Cambodia. Emerg Infect Dis. 2018;24(8):1516–9.	Popovici J, Vantaux A, Primault L, Samreth R, Piv EP, Bin S, et al.	antimalarial-yes, K13 information-no, clinical study/invivo study-yes, Asia-yes, other-p.vivax study
112	29798745	Therapeutic and Transmission-Blocking Efficacy of Dihydroartemisinin/Piperaquine and Chloroquine against Plasmodium vivax Malaria, Cambodia. Emerg Infect Dis. 2018;24(8):1516–9.	Popovici J, Vantaux A, Primault L, Samreth R, Piv EP, Bin S, et al.	antimalarial-yes, K13 information-no, clinical study/invivo study-yes, Asia-yes, other-p.vivax study
113	29703425	Effect of generalised access to early diagnosis and treatment and targeted mass drug administration on Plasmodium falciparum malaria in Eastern Myanmar: an observational study of a regional elimination programme. Lancet. 2018 May 12;391(10133):1916–26.	Landier J, Parker DM, Thu AM, Lwin KM, Delmas G, Nosten FH, et al.	antimalarial-yes, K13 information-yes, clinical study/invivo study-yes, Asia-yes, other-no
114	29690890	A novel field-based molecular assay to detect validated artemisinin-resistant k13	Vachot-Ganée L, Khim N, Iannello A, Legrand E, Kim S, Eam R, et al.	antimalarial-yes, K13 information-yes, clinical study/invivo

		mutants. Malar J. 2018 Apr;17(1):175.		study-no, Asia-yes, other-diagnostic study
115	29676250	Plasmodium falciparum resistance to artemisinin-based combination therapies: A sword of Damocles in the path toward malaria elimination. Parasite. 2018;25:24.	Ouji M, Augereau J-M, Paloque L, Benoit-Vical F.	antimalarial-yes, K13 information-yes, clinical study/invivo study-no, Asia-yes, other-review
116	29659945	Plasmodium falciparum Falcipain-2a Polymorphisms in Southeast Asia and Their Association With Artemisinin Resistance. J Infect Dis. 2018 Jul;218(3):434–42.	Siddiqui FA, Cabrera M, Wang M, Brashear A, Kemirembe K, Wang Z, et al.	antimalarial-yes, K13 information-no, clinical study/invivo study-yes, Asia-yes, other-no
117	29615130	New endoperoxides highly active in vivo and in vitro against artemisinin-resistant Plasmodium falciparum. Malar J. 2018 Apr;17(1):145.	Lobo L, Cabral LIL, Sena MI, Guerreiro B, Rodrigues AS, de Andrade-Neto VF, et al. Plasmodium falciparum. Malar J. 2018 Apr;17(1):145.	antimalarial-yes, K13 information-yes, clinical study/invivo study-no, Asia-no, other-new therapeutic study
118	29615130	New endoperoxides highly active in vivo and in vitro against artemisinin-resistant Plasmodium falciparum. Malar J. 2018 Apr;17(1):145.	Lobo L, Cabral LIL, Sena MI, Guerreiro B, Rodrigues AS, de Andrade-Neto VF, et al. Plasmodium falciparum. Malar J. 2018 Apr;17(1):145.	antimalarial-yes, K13 information-yes, clinical study/invivo study-no, Asia-no, other-new therapeutic study
119	29566683	Population pharmacokinetic and pharmacodynamic properties of artesunate in patients with artemisinin sensitive and resistant infections in Southern Myanmar. Malar J. 2018 Mar;17(1):126.	Lohy Das JP, Kyaw MP, Nyunt MH, Chit K, Aye KH, Aye MM, et al.	antimalarial-yes, K13 information-no, clinical study/invivo study-yes, Asia-yes, other-PKPD study
120	29563721	Suspected Artesunate Resistant Malaria in South India. Vol. 10, Journal of global infectious diseases. 2018. p. 26–7.	Akunuri S, Shraddha P, Palli V, MuraliSantosh B.	antimalarial-yes, K13 information-no, clinical study/invivo study-yes, Asia-yes, other-case reports without K13 markers evaluation
121	29538461	Oxidative stress and protein damage responses mediate artemisinin resistance in	Rocamora F, Zhu L, Liong KY, Dondorp A, Miotto O, Mok S, et al.	antimalarial-yes, K13 information-yes, clinical study/invivo

		malaria parasites. PLoS Pathog. 2018 Mar;14(3):e1006930.		study-no, Asia-no, other-no
122	29535546	Multidrug-resistant malaria and the impact of mass drug administration. Infect Drug Resist. 2018;11:299–306.	Zuber JA, Takala-Harrison S.	antimalarial-yes, K13 information-no, clinical study/invivo study-no, Asia-yes, other-no
123	29512604	In vitro susceptibility of Indian Plasmodium falciparum isolates to different antimalarial drugs & antibiotics. Indian J Med Res. 2017 Nov;146(5):622–8.	Agarwal P, Anvikar AR, Pillai CR, Srivastava K.	antimalarial-yes, K13 information-no, clinical study/invivo study-yes, Asia-yes, other-PKPD study
124	29398391	Origins of the current outbreak of multidrug-resistant malaria in southeast Asia: a retrospective genetic study. Lancet Infect Dis. 2018 Mar;18(3):337–45.	Amato R, Pearson RD, Almagro-Garcia J, Amaratunga C, Lim P, Suon S, et al.	antimalarial-yes, K13 information-yes, clinical study/invivo study-no, Asia-yes, other-review
125	29355852	Drug resistance in Plasmodium. Nat Rev Microbiol. 2018 Mar;16(3):156–70.	Haldar K, Bhattacharjee S, Safeukui I.	antimalarial-yes, K13 information-yes, clinical study/invivo study-no, Asia-no, other-no
126	29318819	Analysis of spatial distribution of artemisinin in Artemisia annua in China. Zhongguo Zhong yao za zhi = Zhongguo zhongyao zazhi = China J Chinese Mater medica. 2017 Nov;42(22):4277–81.	Zhang X-B, Guo L-P, Qiu Z-D, Qu X-B, Wang H, Jing Z-X, et al.	antimalarial-yes, K13 information-no, clinical study/invivo study-no, Asia-yes, other-no
127	29258508	Functional analysis of Plasmodium falciparum subpopulations associated with artemisinin resistance in Cambodia. Malar J. 2017 Dec;16(1):493.	Dwivedi A, Reynes C, Kuehn A, Roche DB, Khim N, Hebrard M, et al.	antimalarial-yes, K13 information-yes, clinical study/invivo study-no, Asia-yes, other-previous samples
128	29192183	Prevalence of mutations linked to antimalarial resistance in Plasmodium falciparum from Chhattisgarh, Central India: A malaria elimination point of view. Sci Rep. 2017 Nov;7(1):16690.	Patel P, Bharti PK, Bansal D, Ali NA, Raman RK, Mohapatra PK, et al.	antimalarial-yes, K13 information-yes, clinical study/invivo study-no, Asia-yes, other-review
129	29177421	Endoperoxide-based compounds: cross-resistance with artemisinins and selection of a Plasmodium falciparum	Paloque L, Witkowski B, Lelièvre J, Ouji M, Ben Haddou T, Ariey F, et al.	antimalarial-yes, K13 information-yes, clinical study/invivo

		lineage with a K13 non-synonymous polymorphism. J Antimicrob Chemother. 2018 Feb;73(2):395–403.		study-no, Asia-yes, other-no
130	29150282	Artesunate-quercetin/luteolin dual drug nanofacilitated synergistic treatment for malaria: A plausible approach to overcome artemisinin combination therapy resistance. Med Hypotheses. 2017 Nov;109:176–80.	Puttappa N, Kumar RS, Yamjala K.	antimalarial-yes, K13 information-no, clinical study/invivo study-no, Asia-no, other-no
131	29132370	Expanding malaria diagnosis and treatment in Lao PDR: lessons learned from a public-private mix initiative. Malar J. 2017 Nov;16(1):460.	Simmalavong N, Phommixay S, Kongmanivong P, Sichanthongthip O, Hongvangthong B, Gopinath D, et al.	antimalarial-yes, K13 information-no, clinical study/invivo study-no, Asia-yes, other-no
132	29078767	Correction to: Prevalence of K13 mutation and Day-3 positive parasitaemia in artemisinin-resistant malaria endemic area of Cambodia: a cross-sectional study. Malar J. 2017 Oct;16(1):435.	Kheang ST, Sovannaroth S, Ek S, Chy S, Chhun P, Mao S, et al.	antimalarial-yes, K13 information-yes, clinical study/invivo study-no, Asia-yes, other-addendum - original paper already included
133	29062913	Safety and effectiveness of mass drug administration to accelerate elimination of artemisinin-resistant falciparum malaria: A pilot trial in four villages of Eastern Myanmar. Wellcome open Res. 2017;2:81.	Landier J, Kajeechiwa L, Thwin MM, Parker DM, Chaumeau V, Wiladphaingern J, et al.	antimalarial-yes, K13 information-yes, clinical study/invivo study-yes, Asia-yes, other-no
134	29020373	Artemether-Lumefantrine Versus Chloroquine for the Treatment of Uncomplicated Plasmodium knowlesi Malaria: An Open-Label Randomized Controlled Trial CAN KNOW. Clin Infect Dis an Off Publ Infect Dis Soc Am. 2018 Jan;66(2):229–36.	Grigg MJ, William T, Barber BE, Rajahram GS, Menon J, Schimann E, et al.	antimalarial-yes, K13 information-no, clinical study/invivo study-yes, Asia-yes, other-no
135	29020247	Lumefantrine Dispersible Tablets in Pediatric Patients With Acute Uncomplicated Plasmodium falciparum Malaria: A Phase 3, Rand. Clin Infect Dis an Off Publ Infect Dis	Toure OA, Mwapasa V, Sagara I, Gaye O, Thompson R, Maheshwar A V, et al.	antimalarial-yes, K13 information-no, clinical study/invivo study-yes, Asia-yes, other-no

		Soc Am. 2017 Oct;65(10):1711–20.		
136	28934435	Declining Transmission and Immunity to Malaria and Emerging Artemisinin Resistance in Thailand: A Longitudinal Study. J Infect Dis. 2017 Sep;216(6):723–31.	Ataíde R, Powell R, Moore K, McLean A, Phyo AP, Nair S, et al.	antimalarial-yes, K13 information-no, clinical study/invivo study-yes, Asia-yes, other-no kelch markers reported
137	28927405	The use of respondent-driven sampling to assess malaria knowledge, treatment-seeking behaviours and preventive practices among mobile and migrant populations in a setting of artemisinin resistance in Western Cambodia. Malar J. 2017 Sep;16(1):378.	Ly P, Thwing J, McGinn C, Quintero CE, Top-Samphor N, Habib N, et al.	antimalarial-yes, K13 information-no, clinical study/invivo study-no, Asia-yes, other-no
138	28895080	Population Pharmacokinetic and Pharmacodynamic Modeling of Artemisinin Resistance in Southeast Asia. AAPS J. 2017 Nov;19(6):1842–54.	Lohy Das J, Dondorp AM, Nosten F, Phyo AP, Hanpithakpong W, Ringwald P, et al.	antimalarial-yes, K13 information-no, clinical study/invivo study-no, Asia-yes, other-modelling study
139	28895080	Population Pharmacokinetic and Pharmacodynamic Modeling of Artemisinin Resistance in Southeast Asia. AAPS J. 2017 Nov;19(6):1842–54.	Lohy Das J, Dondorp AM, Nosten F, Phyo AP, Hanpithakpong W, Ringwald P, et al.	antimalarial-yes, K13 information-no, clinical study/invivo study-no, Asia-yes, other-modelling study
140	28854635	Partner-Drug Resistance and Population Substructuring of Artemisinin-Resistant Plasmodium falciparum in Cambodia. Genome Biol Evol. 2017 Jun 1;9(6):1673–86.	Parobek CM, Parr JB, Brazeau NF, Lon C, Chaorattanakawee S, Gosi P, et al.	antimalarial-yes, K13 information-no, clinical study/invivo study-yes, Asia-yes, other-no
141	28797235	Polymorphisms of Plasmodium falciparum k13-propeller gene among migrant workers returning to Henan Province, China from Africa. BMC Infect Dis. 2017;17(1):560.	Yang C, Zhang H, Zhou R, Qian D, Liu Y, Zhao Y, et al.	antimalarial-yes, K13 information-yes, clinical study/invivo study-no, Asia-yes, other-returning travellers from africa
142	28777791	Antimalarial drug resistance: linking Plasmodium falciparum parasite biology to the clinic. Nat Med. 2017 Aug;23(8):917–28.	Blasco B, Leroy D, Fidock DA.	antimalarial-yes, K13 information-yes, clinical study/invivo study-no, Asia-yes, other-review

143	28711439	Updates on k13 mutant alleles for artemisinin resistance in Plasmodium falciparum. J Microbiol Immunol Infect. 2018 Apr;51(2):159–65.	Zaw MT, Emran NA, Lin Z.	antimalarial-yes, K13 information-yes, clinical study/invivo study-no, Asia-yes, other-review
144	28537265	A tetraoxane-based antimalarial drug candidate that overcomes PfK13-C580Y dependent artemisinin resistance. Nat Commun. 2017 May;8:15159.	O'Neill PM, Amewu RK, Charman SA, Sabbani S, Gnädig NF, Straimer J, et al.	antimalarial-yes, K13 information-no, clinical study/invivo study-no, Asia-yes, other-PKPD
145	28533179	Polymorphisms in pfdhfr and pfdhps genes after five years of artemisinin combination therapy (ACT) implementation from urban Kolkata, India. Infect Genet Evol J Mol Epidemiol Evol Genet Infect Dis. 2017 Sep;53:155–9.	Chatterjee M, Ganguly S, Saha P, Guha SK, Maji AK.	antimalarial-yes, K13 information-no, clinical study/invivo study-yes, Asia-yes, other-no
146	28473165	Unpacking "Artemisinin Resistance". Trends Pharmacol Sci. 2017 Jun;38(6):506–11.	Wang J, Xu C, Lun Z-R, Meshnick SR.	antimalarial-yes, K13 information-yes, clinical study/invivo study-no, Asia-yes, other-review
147	28454557	Longitudinal genomic surveillance of Plasmodium falciparum malaria parasites reveals complex genomic architecture of emerging artemisinin resistance. Genome Biol. 2017 Apr;18(1):78.	Cerqueira GC, Cheeseman IH, Schaffner SF, Nair S, McDew-White M, Phyo AP, et al.	antimalarial-yes, K13 information-yes, clinical study/invivo study-no, Asia-yes, other-retrospective samples aready added from other studies
148	28438194	Malaria profiles and challenges in artemisinin resistance containment in Myanmar. Infect Dis poverty. 2017 Apr;6(1):76.	Nwe TW, Oo T, Wai KT, Zhou S, van Griensven J, Chinnakali P, et al.	antimalarial-yes, K13 information-no, clinical study/invivo study-yes, Asia-yes, other-no
149	28438155	The malaria testing and treatment landscape in the southern Lao People's Democratic Republic (PDR). Malar J. 2017 Apr;16(1):169.	Phanalasy S.	antimalarial-yes, K13 information-no, clinical study/invivo study-no, Asia-yes, other-no
150	28438145	Insights into the availability and distribution of oral artemisinin monotherapy in Myanmar: evidence from a nationally representative outlet survey. Malar J. 2017 Apr;16(1):170.	Thein ST, Khin HSS, Thi A.	antimalarial-yes, K13 information-no, clinical study/invivo study-no, Asia-yes, other-no

151	28410610	An intricate case of multidrug resistant Plasmodium falciparum isolate imported from Cambodia. Malar J. 2017 Apr;16(1):149.	Dell'Acqua R, Fabrizio C, Di Gennaro F, Lo Caputo S, Saracino A, Menegon M, et al.	antimalarial-yes, K13 information-yes, clinical study/invivo study-yes, Asia-no, other-imported case to italy
152	28221121	Molecular Evidence of Drug Resistance in Asymptomatic Malaria Infections, Myanmar, 2015. Emerg Infect Dis. 2017;23(3):517.	Nyunt MH, Shein T, Zaw NN, Han SS, Muh F, Lee S-K, et al.	antimalarial-yes, K13 information-no, clinical study/invivo study-yes, Asia-yes, other-no
153	33319728	Case Report: The First Case of Genotypically Confirmed K13 Propeller Mutation in Sri Lanka and Its Implications on the Elimination Status of Malaria. Am J Trop Med Hyg. 2020 Dec;104(3):964–7.	Fernando D, Weerasekera CJ, Gunasekera WMKT de AW, Hapuarachchi HC, Koo C, Munas M, et al.	antimalarial-yes, K13 information-yes, clinical study/invivo study-yes, Asia-no, other-imported case
154	32679084	Molecular epidemiology of resistance to antimalarial drugs in the Greater Mekong subregion: an observational study. Lancet Infect Dis. 2020 Jul 14;	Imwong M, Dhorda M, Myo Tun K, Thu AM, Phyo AP, Proux S, et al.	antimalarial-yes, K13 information-yes, clinical study/invivo study-no, Asia-yes, other-samples already included from other studies
155	33060063	Triple Artemisinin-Based Combination Therapies for Malaria - A New Paradigm? Trends Parasitol. 2021 Jan;37(1):15–24.	van der Pluijm RW, Amaratunga C, Dhorda M, Dondorp AM.	antimalarial-yes, K13 information-yes, clinical study/invivo study-no, Asia-yes, other-review
156	33139275	Transmission of Artemisinin-Resistant Malaria Parasites to Mosquitoes under Antimalarial Drug Pressure. Antimicrob Agents Chemother. 2020 Dec;65(1).	Witmer K, Dahalan FA, Delves MJ, Yahiya S, Watson OJ, Straschil U, et al.	antimalarial-yes, K13 information-yes, clinical study/invivo study-no, Asia-no, other-review
157	33271239	In vitro reduction of Plasmodium falciparum gametocytes: Artemisia spp. tea infusions vs. artemisinin. J Ethnopharmacol. 2021 Mar;268:113638.	Snider D, Weathers PJ.	antimalarial-yes, K13 information-no, clinical study/invivo study-no, Asia-yes, other-no
158	33370279	Genetic background and PfKelch13 affect artemisinin susceptibility of PfCoronin mutants in Plasmodium	Sharma AI, Shin SH, Bopp S, Volkman SK, Hartl DL, Wirth DF.	antimalarial-yes, K13 information-yes, clinical study/invivo study-no, Asia-yes, other-no

		falciparum. PLoS Genet. 2020 Dec;16(12):e1009266.		
159	33690638	In vitro growth competition experiments that suggest consequences of the substandard artemisinin epidemic that may be accelerating drug resistance in P. falciparum malaria. PLoS One. 2021;16(3):e0248057.	Hassett MR, Roepe PD.	antimalarial-yes, K13 information-yes, clinical study/invivo study-no, Asia-yes, other-no
160	28289248	Antimalarial Drug Resistance: A Threat to Malaria Elimination. Cold Spring Harb Perspect Med. 2017 Jul;7(7).	Menard D, Dondorp A.	antimalarial-no, K13 information-no, clinical study/invivo study-no, Asia-no, other-no
161	28187990	How to Contain Artemisinin- and Multidrug-Resistant Falciparum Malaria. Trends Parasitol. 2017 May;33(5):353–63.	Dondorp AM, Smithuis FM, Woodrow C, Seidlein L von.	antimalarial-no, K13 information-no, clinical study/invivo study-no, Asia-no, other-no
162	33764971	Novel anti-malarial drug strategies to prevent artemisinin partner drug resistance: A model-based analysis. PLoS Comput Biol. 2021 Mar;17(3):e1008850.	Kunkel A, White M, Piola P.	antimalarial-yes, K13 information-no, clinical study/invivo study-no, Asia-no, other-modelling studyy
163	33197753	Evidence for linkage of pfmdr1, pfcrt, and pfk13 polymorphisms to lumefantrine and mefloquine susceptibilities in a Plasmodium falciparum cross. Int J Parasitol Drugs drug Resist. 2020 Dec;14:208–17.	Windle ST, Lane KD, Gadalla NB, Liu A, Mu J, Caleon RL, et al.	antimalarial-yes, K13 information-yes, clinical study/invivo study-no, Asia-no, other-genetic cross study
164	31563454	Importance of kelch 13 C580Y mutation in the studies of artemisinin resistance in Plasmodium falciparum in Greater Mekong Subregion. J Microbiol Immunol Infect. 2020 Oct;53(5):676–81.	Zaw MT, Lin Z, Emran NA.	antimalarial-yes, K13 information-yes, clinical study/invivo study-no, Asia-yes, other-no
